# Evaluation of Novel Chalcone-Thiosemicarbazones Derivatives as Potential Anti-*Leishmania amazonensis* Agents and Its HSA Binding Studies

**DOI:** 10.3390/biom9110643

**Published:** 2019-10-23

**Authors:** Edinéia Pastro Mendes, Carla Marins Goulart, Otávio Augusto Chaves, Viviane dos S. Faiões, Marilene M. Canto-Carvalho, Gerzia C. Machado, Eduardo Caio Torres-Santos, Aurea Echevarria

**Affiliations:** 1 Instituto de Química, Departamento de Química Orgânica, Universidade Federal Rural do Rio de Janeiro, Seropédica 23.890-000, Rio de Janeiro, Brazil; edinea@bio.fiocruz.br (E.P.M.); cmarinsgoulart@gmail.com (C.M.G.); 2 Programa de Pós-graduação em Ciência, Tecnologia e Inovação em Agropecuária, Universidade Federal Rural do Rio de Janeiro, Seropédica, Seropédica 23890-000, Rio de Janeiro, Brazil; 3 Instituto SENAI de Inovação em Química Verde, Maracanã 20271-030, Rio de Janeiro, Brazil; oachaves@firjan.com.br; 4 Laboratório de Bioquímica de Tripanosomatídeos, Instituto Oswaldo Cruz, FIOCRUZ, Rio de Janeiro 21040-900, Brazil; faioes@gmail.com (V.d.S.F.); mcantoca@ioc.fiocruz.br (M.M.C.-C.); machado@ioc.fiocruz.br (G.C.M.)

**Keywords:** *Leishmania amazonensis*, promastigotes, intracellular amastigotes, chalcone-thiosemicarbazones, human serum albumin, spectroscopy, molecular docking

## Abstract

A series of seven chalcone-thiosemicarbazones (**5a**–**5g**) were synthesized and evaluated as potential new drugs (anti-leishmanial effect). Although four of the chalcone-thiosemicarbazones are already known, none of them or any compound in this class has been previously investigated for their effects on parasites of the *Leishmania* genus. The compounds were prepared in satisfactory yields (40–75%) and these compounds were evaluated against promastigotes, axenic amastigotes and intracellular amastigotes of *L. amazonensis* after 48 h of culture. The half maximal inhibitory concentration (IC_50_) values of the intracellular amastigotes were determined to be in the range of 3.40 to 5.95 µM for all compounds assayed. The selectivity index showed value of 15.05 for **5a**, whereas pentamidine (reference drug) was more toxic in our model (SI = 2.32). Furthermore, to understand the preliminary relationship between the anti-leishmanial activity of the chalcone-thiosemicarbazones, their electronic (σ), steric (MR) and lipophilicity (π) properties were correlated, and the results indicated that moieties with electronic withdrawing effects increase the anti-leishmanial activity. The preliminary pharmacokinetic evaluation of one of the most active compound (**5e**) was studied via interaction to human serum albumin (HSA) using multiple spectroscopic techniques combined with molecular docking. The results of antiparasitic effects against *L. amazonensis* revealed the chalcone-thiosemicarbazone class to be novel prototypes for drug development against leishmaniasis.

## 1. Introduction

Leishmaniasis is caused by more than 20 *Leishmania* species belonging to the Trypanosomatidae family. These parasites are transmitted to humans by the bite of female sand flies. There are three principal forms of the disease: cutaneous, mucocutaneous and visceral. This disease is prevalent in 98 countries on five continents and is estimated to cause 700,000 to 1 million new cases and 20,000 to 30,000 deaths annually [1].

The chemotherapeutic treatments for leishmaniasis remain not satisfactory due to their limited effectiveness, long timeline of treatment, high cost and severe secondary effects [2,3]. The first-choice treatment involves the use of pentavalent antimonials (SbV) such as sodium stibogluconate (Pentostam^®^) and meglumine antimoniate (Glucantime^®^) [4]. The second choice includes pentamidine and amphotericin B, which are highly toxic and cause serious side effects [5,6]. More recently, the oral drug miltefosine has been used for the treatment of visceral leishmaniasis, but it presents a high cost and a low therapeutic ratio and, in addition, has mild-to-severe gastrointestinal side effects and teratogenicity [7].

Thus, due to these serious disadvantages of the current treatment, the development of safer and more effective drugs for the treatment of the leishmaniasis is a priority. Currently, our research group has been developing novel compounds based on natural products, such as chalcones and their derivatives.

Chalcones are an important class of natural products with widespread biological activities [8,9,10,11]. This molecular class is present in several vegetal species, and its synthesis is easy, representing a great opportunity to generate several new derivatives. Chalcones have the ability to coordinate metals and exhibit a diversified range of biological activities, e.g., antiparasitic [8,9], anticancer [10] and antioxidant activity [11]. The thiosemicarbazones derivatives also present interesting structural properties due to their several pharmacological effects such as antiparasitic [12,13,14], antimicrobial [15,16] and anticancer activities [17,18].

The molecular composition from hybridizing chalcones and thiosemicarbazones may result in new and interesting molecules with favorable biological activities. In the last few years, some chalcone-thiosemicarbazones have been reported with promising biological activities, such as anticancer [19] and anti-tyrosinase activities [20]. Furthermore, several metal complexes of chalcone-thiosemicarbazones with biological activity have been reported [21,22].

Studies on molecular interactions of drugs with macromolecules have significantly contributed to the understanding of preliminary pharmacodynamics and/or pharmacokinetics evaluation of potential drugs. According to this, human serum albumin (HSA) is the most abundant globular protein in the human bloodstream, having attracted great interest from the pharmaceutical industry because it can bind to a remarkable variety of endogenous and exogenous molecules, impacting their delivery and efficacy and altering mainly their pharmacokinetic properties [23,24]. The HSA is a globular protein consisting of a single polypeptide chain of 585 amino acid residues and is composed of three structurally similar domains (I, II and III). The amino acid residue Trp-214 is the main fluorophore of HSA and generally its fluorescence is used to obtain parameters which are related to the binding ability [25,26].

**Figure 1:**
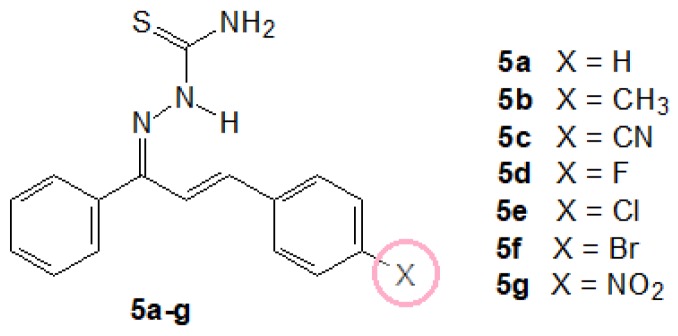
Synthetic hybrid chalcone-thiosemicarbazone structures under study.

Based on the background described above, the main goal of the present work was the synthesis and characterization of a novel series of seven chalcone-thiosemicarbazones: 1-phenyl-3-(4-X-phenyl)-2-ylprop-2-en-1-one thiosemicarbazones, where X=H (**5a**), CH_3_ (**5b**), CN (**5c**), F (**5d**), Br (**5e**), Cl (**5f**) and NO_2_ (**5g**) (Figure 1), as well as the biological evaluation of each compound toward promastigotes, axenic and intracellular amastigotes of *Leishmania amazonensis*. For the compound which presented the best biological activity, its preliminary pharmacokinetic parameter was evaluated via ability to interact to HSA (spectroscopic and theoretical methods).

## 2. Materials and Methods

### 2.1. Organic Synthesis

#### 2.1.1. Chemicals and Instruments

All reagents (hydrochloric acid, acetophenone, X-benzaldehydes, NaOH) and solvents (DMSO-d_6_, ethanol, hexane, ethyl acetate)used in the organic synthesis were purchased from Sigma Aldrich, St. Louis, MO, USA and used without further purification. The Nuclear Magnetic Resonance (NMR) spectra (^1^H and ^13^C) were obtained using a Bruker NMR Ultrashield 400 MHz spectrometer with tetramethylsilane as internal reference and deuterated dimethylsulfoxide (DMSO-d_6_) as the solvent; the chemical shifts were reported in ppm. The infrared (IR) spectra were recorded on a Bruker Vertex 70 spectrometer using potassium bromide (KBr) tablets. Elemental analyses were performed on a PerkinElmer 2400 CHN in the Laboratory of Environmental Science in the State University of Northern Rio de Janeiro (UENF). The melting points were recorded on a Gehaka (PF 1500 Farma) capillary melting points apparatus and are uncorrected. Reactions were monitored by thin layer chromatography (TLC) on Merck silica gel 60 F245 aluminium sheets. TLC spots were visualized by inspection of the plates under ultraviolet (UV) light (254 and 365 nm).

#### 2.1.2. (2*E*)-3-(4-X-phenyl)-1-phenylprop-2-en-1-one (**3a**–**g**)

**Figure 2:**
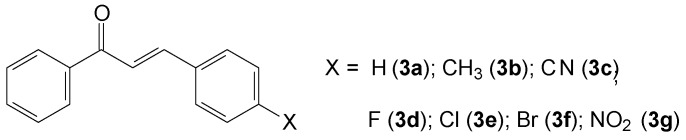
Chemical structure for the synthetic chalcones **3a**–**g**.

The chalcone derivatives (**3a**–**g**, Figure 2) were synthesized and characterized according to the literature reported [27,28].

#### 2.1.3. General Procedure for the Preparation of the (1*E*,2*E*)-3-(4-X-phenyl)-1-phenylprop-2-en-1-one Thiosemicarbazones (**5a**–**g**)

**Figure 3:**
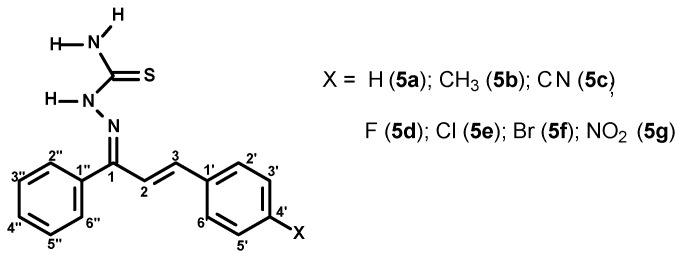
Chemical structure for the synthetic chalcones **3a**–**g**.

A mixture of chalcones (**3a**–**g**) (2 mmol) and thiosemicarbazide (**4**) (2.4 mmol) in hot ethanol (50 mL) had a few drops of concentrated hydrochloric acid added. The reaction mixture was stirred at reflux temperature for 2–6 h, and monitored by TLC using hexane:ethyl acetate (8:2) as the eluent. Afterwards, the precipitate was filtered off and the crude product purified by recrystallization from ethanol, resulting in the target compounds (**5a**–**g**, Figure 3).

##### (1*E*,2*E*)-3-(phenyl)-1-phenylprop-2-en-1-one thiosemicarbazone (**5a**)

Pale yellow amorphous solid; yield 82%; m.p. 133-135 °C (135–137 °C [29]). IR (KBr, cm^−1^): 3469, 3287, 3187 and 3140 (N-H), 3036 (C-H), 1585 (C=N), 1498 and 1444 (C=C), 1128 (C=S), 968 (C=C *trans*-substituted). ^1^H NMR (DMSO-d_6_) δ: 8.65 and 8.50 (2H, 2s, NH_2_), 8.16 (1H, s, NH), 7.67–7.60 (3H, m, H-3″, H-4″ and H-5″), 7.47–7.46 (2H, d, *J* = 7.0 Hz, H-2″, H-6″), 7.38–7.29 (5H, m, H-2′, H-3′, H-4′, H-5′ and H-6′), 7.20–7.17 (1H, d, *J* = 16.0 Hz, H-3), 6.45–6.42 (1H, d, *J* = 16.0 Hz, H-2). ^13^C NMR (DMSO-d_6_) δ: 179.3 (C=S), 148.6 (C=N), 140.1 (C-3), 137.3 (C-1″), 136.2 (C-1′), 130.2 (C-4″), 129.7 (C-2″ and C-6″), 129.1 (C-2′ and C-6′), 128.7 (C-3′ and C-5′), 128.4 (C-3″ and C-5″), 127.3 (C-4′), 119.2 (C-2).

##### (1*E*,2*E*)-3-(4′-methylphenyl)-1-phenylprop-2-en-1-one thiosemicarbazone (**5b**)

Yellow amorphous solid; yield 51%; m.p. 138–140 °C (145 °C [29]). IR (KBr, cm^−1^): 3408, 3341, 3233 and 3143 (N-H), 3037 (C-H), 1598 (C=N), 1477 and 1440 (C=C), 1126 (C=S), 966 (C=C *trans*-substituted). ^1^H NMR (DMSO-d_6_) δ: 11.08 and 8.10 (1H, s, NH), 8.59 and 8.44 (2H, 2s, NH_2_), 7.67–7.62 (3H, m, H-3″, H-4″ and H-5″), 7.37–7.34 (2H, dd, *J* = 7.5 and 1.5 Hz, H-2″ and H-6″), 7.23–7.22 (2H, d, *J* = 8.0 Hz, H-2′ and H-6′), 7.18–7.17 (2H, d, *J* = 8.0 Hz, H-3′ and H-5′), 7.14–7.10 (1H, d, *J* = 16.4 Hz, H-3), 6.42–6.39 (1H, d, *J* = 16.4 Hz, H-2), 2.30 (3H, s, CH_3_). ^13^C NMR (DMSO-d_6_) δ: 179.3 (C=S), 148.9 (C=N), 140.1 (C-3), 139.6 (C-1′), 137.4 (C-1″), 133.6 (C-4′), 129.7 (C-2″ and C-6″), 129.5 (C-2′ and C-6′), 128.7 (C-3″ and C-5″), 128.4 (C-3′ and C-5′), 127.3 (C-4″), 118.1 (C-2), 21.5 (CH_3_).

##### (1*E*,2*E*)-3-(4′-cyanophenyl)-1-phenylprop-2-en-1-one thiosemicarbazone (**5c**)

Pale yellow amorphous solid; yield 75%; m.p. 195–196 °C. IR (KBr, cm^−1^): 3422, 3341, 3246 and 3146 (N-H), 3040 (C-H), 2233 (C≡N), 1599 (C=N), 1549 and 1476 (C=C), 1131 (C=S), 951 (C=C *trans*-substituted). ^1^H NMR (DMSO-d_6_) δ: 8.72 and 8.67 (2H, 2s, NH_2_), 8.19 (1H, s, NH), 7.83–7.81 (2H, d, *J* = 8.5 Hz, H-3′ and H-5′), 7.70–7.68 (2H, d, *J* = 8.5 Hz, H-2′ and H-6′), 7.67–7.62 (3H, m, H-3″, H-4″ and H-5″), 7.38–7.36 (2H, dd, *J* = 7.3 and 1.7 Hz, H-2″ and H-6″), 7.35–7.31 (1H, d, *J* = 16.5 Hz, H-3), 6.56–6.52 (1H, d, *J* = 16.5 Hz, H-2). ^13^C NMR (DMSO-d_6_) δ: 178.4 (C=S), 150.1 (C=N), 140.9 (C-1′), 134.7 (C-3), 133.2 (C-3′ and C-5′), 132.3 (C-2), 130.5 (C-4″), 130.5 (C-1″), 130.3 (C-2″ and C-6″), 128.7 (C-3″ and C-5″), 128.0 (C-2′ and C-6′), 119.2 (CN), 111.0 (C-4′). Anal. Calcd. for C_17_H_14_N_4_S: C, 66.64, H, 4.61, N, 18.29. Found: C, 66.68, H, 4.57, N, 18.32.

##### (1*E*,2*E*)-3-(4′-fluorphenyl)-1-phenylprop-2-en-1-one thiosemicarbazone (**5d**)

Yellow amorphous solid; yield 37%; m.p. 152–154 °C (without m.p., [29]). IR (KBr, cm^−1^): 3413, 3342, 3256 and 3154 (N-H), 3043 (C-H), 1598 (C=N), 1476 and 1443 (C=C), 1229 (C-F), 1127 (C=S), 967 (C=C *trans*-substituted). ^1^H NMR (DMSO-d_6_) δ: 8.63 and 8.50 (2H, 2s, NH_2_), 8.12 (1H, s, NH), 7.68–7.62 (3H, m, H-3″, H-4″ and H-5″), 7.57–7.53 (2H, dd, *J* = 8.8 and 3.2 Hz, H-2′ and H-6′), 7.37–7.34 (2H, dd, *J* = 8.1 and 1.5 Hz, H-2″ and H-6″), 7.23–7.18 (2H, t, *J* = 8.8 Hz, H-3′ and H-5′), 7.15–7.11 (1H, d, *J* = 16.4 Hz, H-3), 6.48–6.44 (1H, d, *J* = 16.4 Hz, H-2). ^13^C NMR (DMSO-d_6_) δ: 179.3 (C=S), 164.1 and 162.1 (1C, d, 1*J* = 248.0 Hz, C-4′), 148.4 (C=N), 138.8 (C-3), 137.2 (C-1″), 132.9 and 132.9 (1C, d, 4*J* = 2.5 Hz, C-1′), 130.6 and 130.5 (2C, d, 3*J* = 8.5 Hz, C-2′ and C-6′), 129.7 (C-2″ and C-6″), 129.5 (C-4″), 128.7 (C-3″ and C-5″), 119.0 (C-2), 116.1 and 115.9 (2C, d, 1*J* = 21.5 Hz, C-3′ and C-5′). Anal. Calcd. for C_16_H_14_FN_3_S: C, 64.19, H, 4.71, N, 10.04. Found: C, 64.25, H, 4.68, N, 10.09.

##### (1*E*,2*E*)-3-(4′-chlorophenyl)-1-phenylprop-2-en-1-one thiosemicarbazone (**5e**)

Pale yellow amorphous solid; yield 57%; m.p. 163-164 °C (164–166 °C [29]). IR (KBr, cm^−1^): 3413, 3341, 3256 and 3154 (N-H), 3043 (C-H), 1598 (C=N), 1476 and 1441 (C=C), 1127 (C=S), 1093 (C-Cl), 968 (C=C *trans*-substituted). ^1^H NMR (DMSO-d_6_) δ: 8.63 and 8.53 (2H, 2s, NH_2_), 8.12 (1H, s, NH), 7.67–7.60 (3H, m, H-3″, H-4″ and H-5″), 7.52–7.50 (2H, d, *J* = 8.5 Hz, H-2′ and H-6′), 7.42–7.41 (2H, d, *J* = 8.5 Hz, H-3′ and H-5′), 7.37–7.35 (2H, dd, *J* = 6.9 and 1.0 Hz, H-2″ and H-6″), 7.20–7.16 (1H, d, *J* = 16.4 Hz, H-3), 6.47–6.44 (1H, d, *J* = 16.4 Hz, H-2). ^13^C NMR (DMSO-d_6_) δ: 178.3 (C=S), 150.7 (C=N), 135.5 (C-3), 135.2 (C-1″), 133.6 (C-1′), 130.6 (C-4′), 130.5 (C-4″), 130.2 (C-2″ and C-6″), 129.6 (C-2), 129.3 (C-2′ and C-6′), 129.0 (C-3′ and C-5′), 128.7 (C-3″ and C-5″).

##### (1*E*,2*E*)-3-(4′-bromophenyl)-1-phenylprop-2-en-1-one thiosemicarbazone (**5f**)

Pale yellow amorphous solid; yield 43%; m.p. 166–168 °C (without m.p. [19]). IR (KBr, cm^−1^): 3414, 3341, 3256 and 3154 (N-H), 3043 (C-H), 1598 (C=N), 1476 and 1441 (C=C), 1127 (C=S), 1072 (C-Br), 969 (C=C *trans*-substituted). ^1^H NMR (DMSO-d_6_) δ: 8.65 and 8.54 (2H, 2s, NH_2_), 8.15 (1H, s, NH), 7.68–7.62 (3H, m, H-3″, H-4″ and H-5″), 7.57–7.55 (2H, d, *J* = 8.5 Hz, H-2′ and H-6′), 7.46–7.44 (2H, d, *J* = 8.5 Hz, H-3′ and H-5′), 7.37–7.35 (2H, dd, *J* = 7.0 and 1.5 Hz, H-2″ and H-6″), 7.22–7.18 (1H, d, *J* = 16.5 Hz, H-3), 6.46–6.41 (1H, d, *J* = 16.5 Hz, H-2). ^13^C NMR (DMSO-d_6_) δ: 178.3 (C=S), 150.7 (C=N), 135.5 (C-3), 135.5 (C-1″), 132.3 (C-3′ and C-5′), 130.6 (C-1′), 130.5 (C-4″), 130.2 (C-2″ and C-6″), 129.6 (C-2), 129.3 (C-2′ and C-6′), 128.7 (C-3″ and C-5″), 122.3 (C-4′). Anal. Calcd. for C_16_H_14_BrN_3_S: C, 53.34, H, 3.92, N, 11.66. Found: C, 53.39, H, 3.88, N, 11.72.

##### (1*E*,2*E*)-3-(4′-nitrophenyl)-1-phenylprop-2-en-1-one thiosemicarbazone (**5g**)

Orange amorphous solid; yield 66%; m.p. 188–190 °C (184-186 °C [30]). IR (KBr, cm^−1^): 3482, 3337, 3249 and 3152 (N-H), 3057 (C-H), 1594 (C=N), 1512 and 1471 (C=C), 1341 (N=O), 1131 (C=S), 966 (C=C *trans*-substituted). ^1^H NMR (DMSO-d_6_) δ: 8.70 and 8.69 (2H, 2s, NH_2_), 8.20–8.19 (3H, m, NH, H-3′ and H-5′), 7.76–7.64 (2H, d, *J* = 8.5 Hz, H-2′ and H-6′), 7.68–7.61 (3H, m, H-3″, H-4″ and H-5″), 7.38–7.35 (3H, m, H-3, H-2″ and H-6″), 6.61–6.58 (1H, d, *J* = 16.4 Hz, H-2). ^13^C NMR (DMSO-d_6_) δ: 179.5 (C=S), 147.7 (C-4′), 147.5 (C=N), 143.1 (C-1′), 137.3 (C-3), 136.9 (C-1″), 129.7 (C-2″ and C-6″), 129.4 (C-3″ and C-5″), 128.8 (C-2′ and C-6′), 128.3 (C-4″), 124.2 (C-3′ and C-5′), 123.4 (C-2).

### 2.2. Biologic Assays

#### 2.2.1. Chemicals

The chalcone-thiosemicarbazones (**5a**-**g**) were solubilized in dimethylsulphoxide (DMSO; Sigma Aldrich, St. Louis, MO, USA) to obtain a stock solution of 10 mM.

#### 2.2.2. Parasite Cultures

*Leishmania amazonensis* (MHOM/BR/77/LTB0016) were regularly isolated from infected mice and maintained in culture as promastigotes through weekly passages in Schneider’s medium (Sigma Aldrich, St. Louis, MO, USA) supplemented with 10% (*v*/*v*) heat-inactivated fetal calf serum, penicillin (100 UI/mL), and streptomycin (100 µg/mL) at 299K. Parasites were maintained until the 10th passage [31]. The Animal Ethics Committee of the Oswaldo Cruz Foundation (license number L026/2015) approved this study.

#### 2.2.3. Promastigote Assays

Promastigotes of *L. amazonensis* (1 × 10^6^ promastigotes/mL) in the 4th day on culture in the conditions described above were incubated with the chalcones serially diluted in culture medium (0.032% v/v DMSO at the highest concentration tested) on a 96-well cell culture plate (TPP™ test plate, Switzerland). The assayed concentrations ranged from 50 to 1.56 µM for all chalcone-thiosemicarbazones (**5a**–**g**) and for pentamidine (Sigma Aldrich, St. Louis, MO, USA). The plates were incubated in the presence of the compounds for 24 h at 299K. All compounds were analyzed in at least three experiments performed in triplicate. Pentamidine was used as the reference drug and diluted in the same concentrations used for **5a**–**g**. The vehicle (DMSO) had no effect on the parasites.

The antileishmanial activity was evaluated by adding 22 µL of 3-(4,5-dimethylthiazol-2-yl)-2-5diphenyltetrazolium bromide (MTT) at 5 mg/mL (Sigma Aldrich, St. Louis, MO, USA) to each well. After 2 h, 80 µL of DMSO was added and the optical density was determined at a wavelength of 570 nm in a microplate reader (µQuant Bio-Tek Instruments^®^, Winooski). The assays were carried out in triplicate in 96-well plates (Costar, New York, USA). The inhibition percentage was estimated by the comparison with the non-treated control. The values for the inhibitory concentration for 50% of the promastigotes (IC_50_) were calculated by a sigmoidal model.

#### 2.2.4. Macrophage Cytotoxicity

Peritoneal macrophages from albino laboratory-bred strain (BALB/c) mice were obtained in RPMI 1640 medium at pH 7.2 (Gibco, New York, NY, USA) supplemented with 10% (v/v) heat-inactivated fetal bovine serum, 100 U/mL penicillin and 0.1 mg/mL of streptomycin and distributed (1 × 10^6^ macrophages/well) in triplicate in 96-well plates (Falcon Co., Franklin Lakes, USA) and incubated for 1 h in a 5% CO_2_ atmosphere incubator at 310K. The supernatant was removed, and the compounds were added to the plate at concentrations from 10 µM to 50 µM. Next, the cultures were incubated for 48 h in a CO_2_ incubator at 310K. After incubation, the supernatant was removed and a resazurin solution (50 µM) was added to each well and incubated at 310K for 4 h. Furthermore, the fluorescence was measured using a wavelength of 560 nm for excitation and 590 nm for detection (Molecular Devices, Silicon Valley, USA). Results were expressed as the percentage of viable cells compared to the control cells treated with the highest DMSO dose used to dissolve the drugs. The 50% cytotoxic concentration (CC_50_) was calculated using GraphPad Prism 6.0 software (GraphPad Company, San Diego, CA, USA).

#### 2.2.5. Intracellular Amastigotes Assays

Peritoneal macrophages were obtained BALB/c mice with cold Roswell Park Memorial Institute (RPMI) 1640 medium (Sigma Aldrich, St. Louis, MO, USA) supplemented as above. The cell suspension was adjusted to 1 × 10^6^ macrophage/mL and incubated in a LAB-TEK 8-chamber slide (Nunc, Roskilde, Denmark) at 310K and 5% CO_2_ for 1 h. Non-adherent cells were removed and stationary-phase *L. amazonensis* promastigotes were added at a 3:1 parasite/macrophage ratio. The cultures were incubated for a further 4 h, and free parasites were removed. The chambers were washed, and the monolayers were incubated with the compounds for 48 h. Infected macrophages incubated only in the culture medium were used as controls to compare with the infected treated macrophages. The concentration of DMSO in the wells did not exceed 0.001% (*v*/*v*), which is not toxic to the parasites. The cultures were fixed in methanol and stained with the hematological system Instant Prov (Newprov^®^, Curitiba, Brazil). The slides were examined by light microscopy, and the number of infected macrophages and the mean number of parasites per macrophage were determined in 100 cells counted. The experiments were performed twice in triplicate. The percentage of infected macrophages was calculated by dividing the number of infected macrophages by the total number of macrophages (infected and uninfected) counted on the slides, multiplied by 100. To determine the infection index, the percentage of infected macrophages was multiplied by the mean number of parasites per macrophage. The percentage inhibition of infection was calculated by multiplying the difference between the infection index of the control and the compound by 100 and dividing by the infection index (IF) of the control using the Equation (1):

IF = % infected cells × amastigotes number/total number of macrophages(1)

The IC_50_ ± SD (SD: standard deviation) values were obtained by a sigmoidal model using Origin 6.0 with statistical error limits up to 10%. All tests were conducted in duplicate for each concentration, and two independent assays were performed.

#### 2.2.6. Axenic Amastigotes Assays

Promastigote forms of *L. amazonensis* (MHOM/BR/77/LTB0016) were grown to a stationary phase, washed once in phosphate buffer solution (PBS) (pH = 7.4) and adjusted to 5 × 10^6^ parasites/mL in Schneider’s medium, supplemented with 20% fetal bovine solution (FBS) pH = 5.5 and maintained at 305K. After 5 days of cultivation, the rounded forms without free flagella were obtained and, after heat shock, were used in the appropriate assays [32]. To evaluate the activity against axenic amastigotes, the parasites were incubated with increasing concentrations of the prototypes (diluted in DMSO) for 72 h at 305K. These assays were performed in triplicate in 96-well flat bottom plates (Falcon Co, Franklin Lakes, USA). The leishmanicidal activity was evaluated by adding 22 μl of resazurin to each well. After 4 h, the cell viability was measured as described above. Calculation of the IC_50_ values was determined in relation to the control by a sigmoidal model using GraphPad Prism 6.0 software.

#### 2.2.7. Statistical Analysis

Significance was determined using a non-paired Student’s *t*-test. Differences were considered significant when *p* < 0.05. Each experiment was, at minimum, performed in triplicate.

### 2.3. HSA Binding Studies

#### 2.3.1. Spectroscopic Analysis

The chemicals HSA and PBS buffer were purchased from Sigma Aldrich, St. Louis, MO, USA and the pH of buffer solution was checked (pH = 7.40). Jasco J-815 optical spectrometer (Jasco Easton, MD, USA) was used as equipment for steady-state fluorescence and circular dichroism (CD) spectra measurements and a thermostatic cuvette holder Jasco PFD-425S15F (Jasco Easton, MD, USA) was applied for control of temperature in the quartz cell (1.0 cm optical path). All spectra were recorded as the average of three scans with appropriate background corrections. For steady-state fluorescence measurements it was applied 305–450 nm range as emission wavelength (λ_exc_ = 295 nm) at 289K, 296K, 303K and 310K. First, it was recorded spectra for HSA solution (1.00 × 10^−5^ M, in PBS) and then it was added manually the compound **5e**, achieving final ligand concentrations of 0.35; 0.70; 1.05; 1.40; 1.74; 2.09; 2.44; and 2.78 × 10^−5^ M.

In order to delete the possible absorption contribution of the compound **5e** in the steady-state fluorescence results, inner filter effect was applied, according to the Equation 2 [33]:

(2) $$F_{cor}=F_{Obs}10^{\left[\frac{Aex+Aem}{2}\right]}$$

where *F_cor_* and *F_obs_* are the corrected and observed fluorescence intensity values, while *A_ex_* and *A_em_* represent the absorbance value at the excitation (λ = 295 nm: ε = 6,235 cm^−1^M^−1^ in PBS) and emission wavelengths (λ = 340 nm: ε = 4,550 cm^−1^M^−1^ in PBS).

The steady-state fluorescence results for the interaction HSA:5e were analyzed according to Stern-Volmer (Equation 3), double logarithmic (Equation 4), van’t Hoff (Equation 5) and Gibbs’ free energy (Equation 6) approximations [25,26]:

(3) $$\frac{F_{0}}{F}=1+K_{sv}\left[Q\right]=1+k_{q}\tau_{0}\left[Q\right]$$

(4) $$log\left(\frac{F_{0}-F}{F}\right)=\log K_{a}+n\log\left[Q\right]$$

(5) $$\ln K_{a}=-\frac{\Delta H{}^{\circ}}{RT}+\frac{\Delta S{}^{\circ}}{R}$$

(6) ΔG°=ΔH°−TΔS°

where *F*_0_ and *F* are the steady-state fluorescence intensities of HSA in the absence and presence of **5e**, respectively. [*Q*], *K_SV_* and *k_q_* are the **5e** concentration, Stern-Volmer quenching constant and bimolecular quenching rate constant, respectively. *τ*_0_ is the experimental fluorescence lifetime of HSA in the absence **5e** (5.98 ± 0.15) × 10^−9^ s. *K_a_* and *ƒ* are the binding constant and fraction of the initial fluorescence intensity corresponding to the fluorophore which is accessible by the quencher (*f* ≈ 1.00), respectively. Δ*H*°, Δ*S*° and Δ*G*° are the enthalpy, entropy and Gibbs’ free energy change, respectively. *T* and *R* are the temperature (289 K, 296 K, 303 K and 310 K) and gas constant (8.3145 Jmol^−1^K^−1^), respectively. All quantitative values were expressed as “± SD” (obtained through three scans).

The fluorimeter model FL920 CD from Edinburgh Instruments (Edinburgh, UK), equipped with an electrically pumped laser (EPL, with λ_exc_ = 280 ± 10 nm; a pulse of 850 ps with the energy of 1.8 µW/pulse; monitoring emission at 340 nm) was used for time-resolved fluorescence decays measurement. The experiment was conducted for a 3.0 mL HSA solution (1.00 × 10^−5^ M, in PBS) without and in the presence of **5e** (2.78 × 10^−5^ M) at room temperature.

The fluorimeter model Xe900 from Edinburgh Instruments (Edinburgh, UK) was used for synchronous fluorescence (SF) measurement in the 240–320 nm range by setting Δλ = 60 nm and Δλ = 15 nm for tryptophan and tyrosine residues, respectively. The experiment was conducted for a 3.0 mL HSA solution (1.00 × 10^−5^ M, in PBS) without and in the presence of successive additions of **5e** (in the same concentration range used in the steady-state fluorescence measurement) at room temperature.

Circular dichroism (CD) spectra were measured in the same equipment used in the steady-state fluorescence analysis, using 200–250 nm range at 310K (human body temperature). The experiment was conducted for a 3.0 mL HSA solution (1.00 × 10^−6^ M, in PBS) in the absence and presence of **5e** (2.78 × 10^−5^ M). Each spectrum obtained was the average of three scans. The intensity of the signal from the CD spectra was expressed as mean residue ellipticity (MRE), defined according to Equation (7) [34]:

(7) $$\mathrm{MRE}=\frac{\theta }{10\times n\times l\times Cp}$$

where *θ*, *n*, *l* and *C_p_* are the observed CD (in millidegrees), number of amino acid residues (585 for HSA) [35], optical pathlength of the cell (1.00 cm) and molar concentration of HSA (1.00 × 10^−6^ M), respectively.

In order to calculate quantitative information on the percentage of changes in the α-helix content upon 5e binding, the resulting MRE at 208 and 222 nm (far-UV region) were analyzed according to Equations (8A) and (8B) [36]:

(8A) $$\alpha -\mathrm{helix}\%=\frac{\left(-\mathrm{MRE}208-4000\right)}{\left(33000-4000\right)}\times 100$$

(8B) $$\alpha -\mathrm{helix}\%=\frac{\left(-\mathrm{MRE}222-2340\right)}{30300}\times 100$$

#### 2.3.2. Molecular Docking Analysis for the Interaction HSA:**5e**

The chemical structure **5e** was built and energy-minimized with Spartan’14 program (Wavefunction, Inc., Irvine, CA, USA). The Density Functional Theory (DFT) method was used, with B3LYP potential and basis set 6-31G* for the minimization of **5e** structure. The crystallographic structure of HSA was obtained in the Protein Data Bank, with access code 1N5U [35]. Molecular docking studies were performed with GOLD 5.6 program (CCDC, Cambridge Crystallographic Data Centre).

The GOLD 5.6 program added the hydrogen atoms to the protein structure according to the ionization and tautomeric states inferred by the program. Molecular docking calculations were explored for the three main protein binding pocket. Thus, docking interaction cavity in the protein was established with a 10 Å radius from the Trp-214, Tyr-411, and Tyr-161 residues, for sites I, II and III, respectively. These amino acid residues were chosen according to the crystallographic structure of each site probe inside HSA (warfarin, ibuprofen and camptothecin) [37,38]. The number of genetic operations (crossover, migration, mutation) in each docking run used in the search procedure was set to 100,000. The scoring function used was ‘ChemPLP’, which is the default function of the GOLD 5.6 program [39]. Figures of the best docking pose were generated with the PyMOL program (DeLano Scientific LLC).

## 3. Results and Discussion

### 3.1. Synthesis of Chalcone-Thiosemicarbazones 

**Scheme 1:**
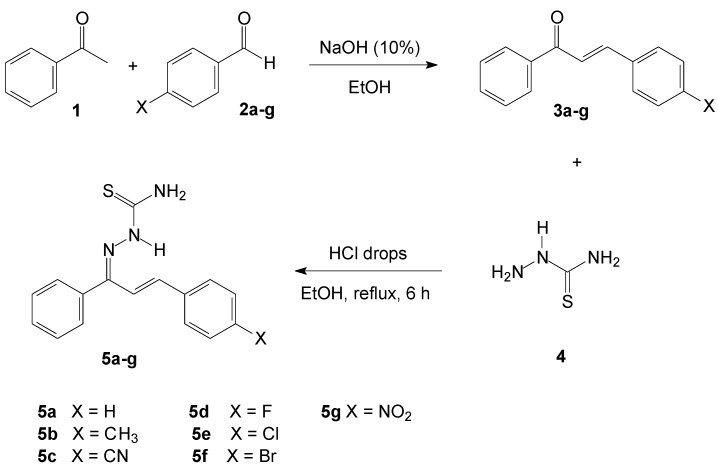
Synthetic pathways for chalcone-thiosemicarbazones.

A series of seven 1-phenyl-3-(4-X-phenyl)-2-ylprop-2-en-1-one thiosemicarbazones (**5a**–**g**) were prepared using an adapted pathway [40] involving substituted chalcones (**3a**–**g**) and thiosemicarbazide (**4**). The substituted chalcones were synthetized by a traditional Claisen-Schmidt condensation reaction [27] using acetophenone (**1**) and X-benzaldehydes (**2a**–**g**) in the presence of NaOH and ethanol. After the purification process with ethanol, it was obtained the synthetic compounds in good yields (75–93%). Then, the chalcone-thiosemicarbazones were obtained from a mixture of previously synthesized substituted chalcones and thiosemicarbazide using a few drops of concentrated HCl and ethanol as a solvent at the reflux temperature within 6 h (Scheme 1). The products were purified by recrystallization from ethanol in reasonable yields (40–75%).

All spectra for the characterization of the synthetic compounds **5a**–**5b** are shown as Figures S1–S19 in the supplementary material. The infrared spectra of the chalcone-thiosemicarbazones showed the disappearance of the carbonyl band (ν C=O) at approximately 1640 cm^−1^, which corresponds to the precursor chalcones [41], and a new band (ν C=N) at 1585–1598 cm^−1^, resulting from imine, was evidenced, being in accordance with literature [22]. Additionally, the ν C=S absorption was observed in the range of 1126–1131 cm^−1^.

**Scheme 2:**
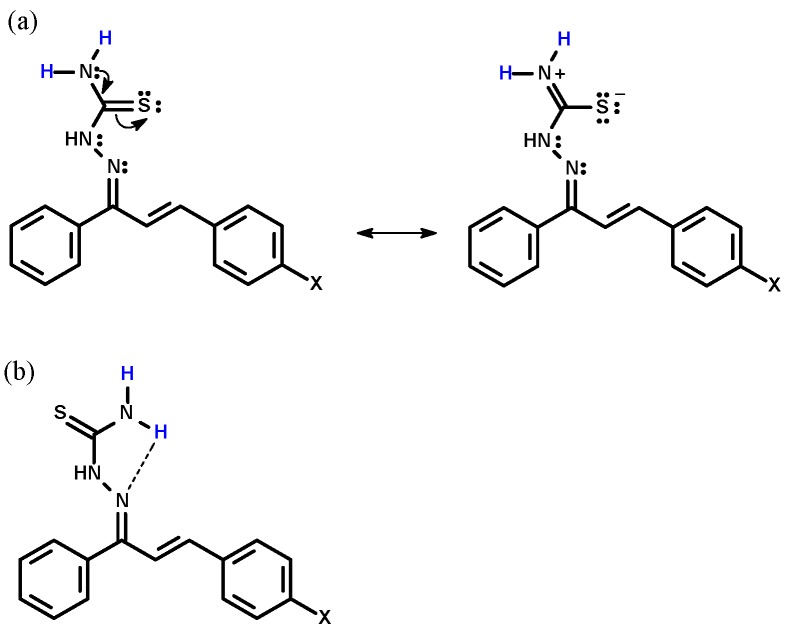
Effects involving the hydrogens H_2_N: (**a**) resonance effect with thiocarbamoyl group; (**b**) intramolecular hydrogen bond between the hydrogen and azomethine nitrogen.

The ^1^H and ^13^C chemical shift values were used to characterize the proposed structures of **5a**–**g** (the spectra are shown in the Supplementary data). The chemical shift values of NH_2_ were absorbed as two singlets in the range of δ 8.50–8.72 due to the resonance effect from the thiocarbamoyl moiety, and the hydrogen bond between one of the hydrogens to a sulphur atom led to diastereotopic hydrogens, as indicated in Scheme 2a,b, respectively. On the other hand, the resonance effect caused by electron acceptor groups in the aromatic ring caused a more upfield absorption in the range of δ 8.12–8.19 as a singlet to HN.

The ^13^C NMR spectra showed the chemical shifts in the typical range of δ 178.3–179.5 assigned to C=S. The chemical shifts of C=N were observed at δ 147.5–150.7 due to the resonance effect with a conjugated double bond enhancing the electronic density of this carbon atom. The other carbon atoms showed chemical shift values as expected.

### 3.2. Anti-Leishmanial Effects 

The treatment of the parasites with the chalcone-thiosemicarbazones **5a**–**g** against *L. amazonensis* promastigotes resulted in a dose-dependent response after 48 h of culture. The values of 50% of growth inhibitory activity (IC_50_) was assessed by the MTT method [42], and the values were obtained by a sigmoidal curve, relating the percentage of growth inhibition and the log of drug concentration in µM, as shown in Table 1. The results revealed a good inhibitory effect, with the exception of **5c** and **5g** which showed no significant activity. The most active compound (**5e**) showed an IC_50_ value of 5.22 ± 0.75 µM, and the positive control pentamidine had an IC_50_ value of 4.90 ± 0.60 µM.

The assays with axenic amastigotes of *L. amazonensis* were performed with **5a**, **5b**, **5d**, **5e** and **5f** over 72 h of culture. The results showed good growth inhibitory effects for all assayed compounds with IC_50_ in the range of 3.19 to 7.08 µM, with the chalcone-thiosemicarbazone chloro-substituted (**5e**) the most active compound. On the other hand, pentamidine showed IC_50_ = 12.29 ± 1.17 µM.

Thus, after the promising results against promastigotes and axenic amastigotes of *L. amazonensis*, the cytotoxicity and activity against intracellular amastigotes were evaluated for **5a**, **5b**, **5d**, **5e** and **5f**. The cytotoxic effects were assessed using murine peritoneal macrophages after 48 h of culture. The pentamidine, used as a positive control, was also assayed under the same conditions. The macrophages in the presence of the chalcone-thiosemicarbazones showed LD_50_ in the range of 32.48 to 56.35 µM, and the pentamidine with LD_50_ = 25.85 ± 4.06 µM.

The treatment of *L. amazonensis*-infected macrophages with **5a**, **5b**, **5d**, **5e** and **5f** after 48 h of culture reduced the infection, with IC_50_ in the range of 3.40 to 5.95 µM, as shown in Table 1, whereas the reduction was 11.12 µM for pentamidine.

Thus, the selectivity index (SI = LD_50_/IC_50_ intracellular amastigotes) showed values in the range of 7.27 and 15.05, whereas pentamidine was found to be more toxic in our model (SI = 2.32). It is important to highlight that some guidelines suggest an SI higher than 10 and IC_50_ against intracellular amastigotes below 10 µM as thresholds for an anti-leishmanial hit before proceeding with further studies [43,44]. The chalcone-thiosemicarbazones **5a** and **5f** met both criteria.

It is interesting that the growth inhibitory effects of chalcone-thiosemicarbazones against *L. amazonensis* axenic and intracellular amastigotes were observed in the same numerical range with lower values when compared to pentamidine but also with a lower toxicity, thus, consequently, it can be considered to be safer.

To understand which parameters are involved in the antileishmanial activity of the chalcone-thiosemicarbazones, their electronic (σ) [45], steric (MR) and lipophilicity (π) properties [46] (see Table 2) were correlated with log IC_50_ or 1/IC_50_ values of *L. amazonensis* axenic amastigotes by linear regression models. After analysis, it was observed that the best correlation was with the electronic parameter (Hammett constant, σ_p_) in the polynomial model with R^2^ = 0.9998 (coefficient of determination), SD = 0.0033 (standard deviation), *n* = 5 and *p* = 0.00024. Thus, this result indicates that moieties with electronic withdrawing effects increase their anti-leishmanial activity.

### 3.3. Evaluation on the Interaction between HSA and ***5e***

**Figure 4:**
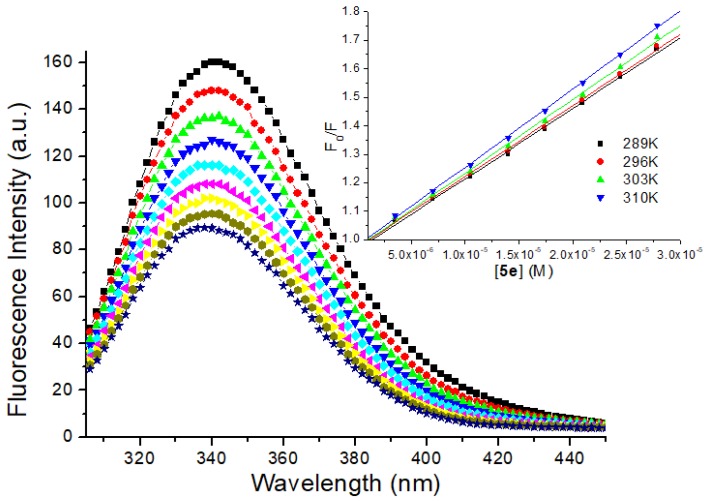
Steady-state fluorescence emission for HSA without and in the presence of successive additions of **5e** at 310K and pH = 7.4. [HSA] = 1.00 × 10^−5^ M and [**5e**] = 0.35; 0.70; 1.05; 1.40; 1.74; 2.09; 2.44; and 2.78 × 10^−5^ M.

Among the synthetic organic compounds which presented the highest toxic activity against *L. amazonensis*, the chalcone-thiosemicarbazone chloro-substituted (**5e**) was selected as a model to study the interaction with the main carrier protein in the human bloodstream (HSA) [35]. The steady-state fluorescence emission of HSA (mainly due to the presence of Trp-214 residue) and its quenching upon **5e** addition is showed in Figure 4. Since, about 50% of HSA fluorescence emission decreased and there is not any significant blue or red shift in the maximum fluorescence emission of the albumin (λ_em_ = 340 nm) upon maximum addition of the ligand (2.78 × 10^−5^ M), there is an indication that **5e** probably can interact with HSA in the region next to Trp-214 residue and does not perturb the microenvironment around the main albumin’s fluorophore [47].

It is very well known that two main different mechanisms account for the interaction fluorophore/quencher (e.g., serum albumin/ligand): dynamic and/or static process. Dynamic and static quenching can be distinguished by Stern-Volmer analysis (inset in the Figure 4), as well as the known relationship between Stern-Volmer quenching constant (*K_SV_*) and bimolecular quenching rate constant (*k_q_*) at different temperatures [47,48]. The calculated *K_SV_* values are practically the same inside the experimental error (Table 3); however, the *k_q_* values are about three orders of magnitude larger than the diffusional collision quenching constant (*k_diff_* ~7.40 × 10^9^ M^−1^s^−1^, according to the Smoluchowski-Stokes-Einstein theory at 298 K) [49], indicating that the potential anti-*Leishmania amazonensis* agent (**5e**) can interact to HSA via a ground state association (static process).

**Figure 5:**
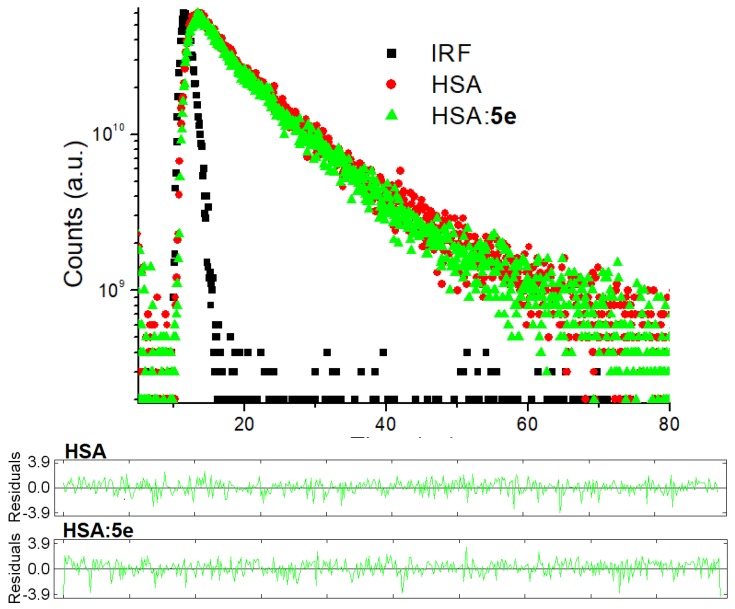
Time-resolved fluorescence decays for human serum albumin (HSA) without and in the presence of **5e** at room temperature and pH = 7.4. [HSA] = 1.00 × 10^−5^ M and [**5e**] = 2.78 × 10^−5^ M. IRF: Instrument response function.

In order to further confirm if this fluorescence quenching mechanism really occurs via a static or dynamic process, time-resolved fluorescence decays were measured for HSA in the absence and presence of **5e** (Figure 5). The HSA solution exhibited two fluorescence lifetimes (*τ*), having the second one the largest contribution (~78.0%): *τ*_1_ = 1.80 ± 0.16 ns and *τ*_2_ = 5.98 ± 0.15 ns **−**χ^2^ = 1.102. These results are in agreement with those in the literature [50]. Upon the maximum addition of the compound **5e** to the protein solution, the new fluorescence lifetimes data are the same inside the experimental error: *τ*_1_ = 1.77 ± 0.12 ns and *τ*_2_ = 5.91 ± 0.16 ns **−**χ^2^ = 1.110. These results are a clear indication that really the static fluorescence process is the main quenching mechanism for the interaction between HSA and **5e [36]**.

The evaluation of binding constant (*K_a_*) between serum albumin and ligands is important to understand the biodistribution of a potential drug in the plasma, body tissues and organs. A too weak binding can lead to a poor distribution of the molecule in the body, while strong binding can decrease the concentrations of free molecules in the bloodstream [24]. For static quenching, the steady-state fluorescence quenching data could be analyzed to calculate the *K_a_* and number of binding sites (*n*) values by double logarithmic approximation (Figure S20 in the supplementary material) [25]. The *K_a_* values for the interaction HSA:**5e** are in the order of 10^4^ M^−1^ (Table 3), which indicates a moderate binding affinity between the potential anti-*Leishmania amazonensis* agent and albumin [24,26]. In addition, *n* ~ 1 indicates that just one **5e** can interact to one main binding site in the albumin structure [48]. The chalcone-thiosemicarbazone chloro-substituted under study showed the same binding trend toward HSA compared to the interaction of some chalcone-thiosemicarbazone derivatives and serum albumin [21].

When small molecules bind to proteins, intermolecular interactions, such as hydrogen bounding, hydrophobic, electrostatic and van der Waals forces, can be involved. The thermodynamic analysis (enthalpy and entropy change − Δ*H*° and Δ*S*°, respectively—calculated according to the van’t Hoff plot in the Figure S21 in the supplementary material) can be applied to identify the main interaction forces [51]. The Δ*S*° > 0 (Table 3) can be related to hydrophobic forces and/or desolvation process and Δ*H*° < 0 (Table 3) can be related to hydrogen bonding and/or electrostatic forces [51]. In addition, the negative Δ*G*° values are consistent with the spontaneity of the binding process in all temperatures under study (Table 3). Since the negative ΔG° value for the complete association process is primarily determined by the positive entropy change accompanying the first step and the negative enthalpy change of the second step, the association HSA:**5e** is enthalpically and entropically driven [52].

### 3.4. Evaluation on the Microenvironment and Structure of HSA upon ***5e*** Binding

**Figure 6:**
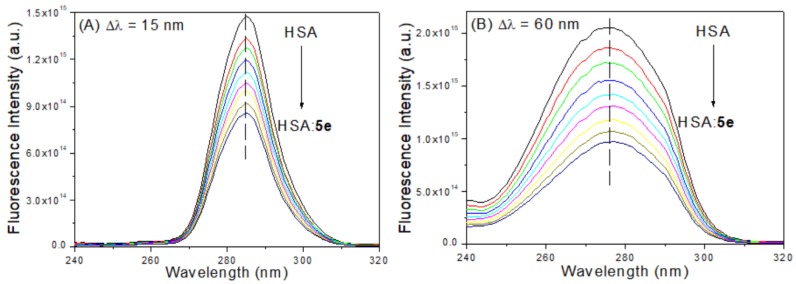
Synchronous fluorescence (SF) spectra for HSA without and in the presence of **5e** at (**A**) Δλ = 15 nm and (**B**) Δλ = 60 nm. [HSA] = 1.00 × 10^−5^ M and [**5e**] = 0.35; 0.70; 1.05; 1.40; 1.74; 2.09; 2.44; and 2.78 × 10^−5^ M.

Synchronous fluorescence (SF) spectroscopy is a common technique used to study the changes in the microenvironment of tyrosine and tryptophan amino acid residues when the difference between excitation and emission wavelength (∆λ) is 15 and 60 nm, respectively [25]. Figure 6 shows the effect of increasing the concentration of **5e** on the synchronous spectra of HSA at ∆λ = 15 nm and ∆λ = 60 nm. In all cases, a decrease in emission intensity of serum albumin with increasing in ligand concentration was observed; however, there is not any significant blue or red shift in the maximum fluorescence emission of albumin upon ligand binding, indicating that **5e** can bind to serum albumin without affect significantly the microenvironment of both tyrosine and tryptophan residues. These results are different from those observed for the interaction between serum albumin and some chalcone-thiosemicarbazone derivatives [21].

**Figure 7:**
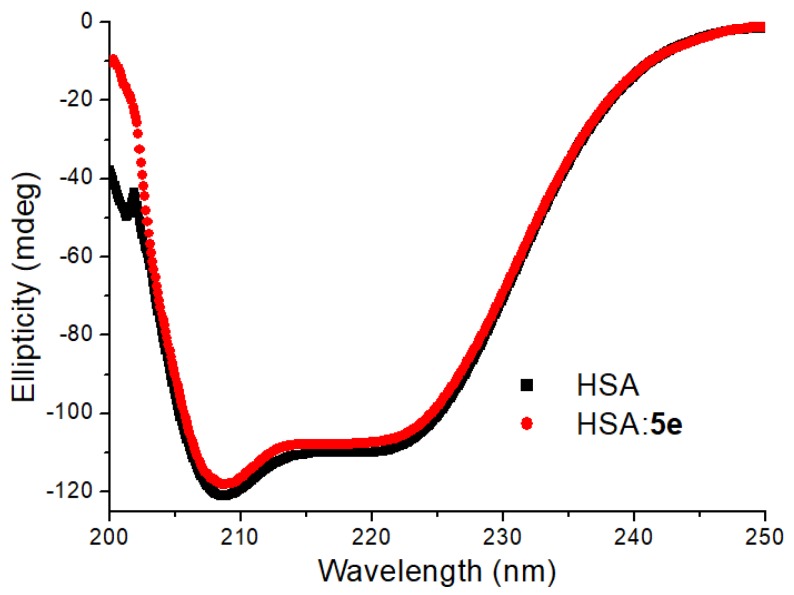
CD spectra for HSA without and in the presence of **5e** at 310K and pH = 7.4. [HSA] = 1.00 × 10^−5^ M and [**5e**] = 2.78 × 10^−5^ M.

Circular dichroism (CD) spectroscopy is a widely technique employed to evaluate the interaction between exogenous agents and macromolecules (e.g., proteins). Thus, CD measurement allows obtaining information on perturbation of the albumin upon ligand binding. More specifically, far-UV CD spectra can offer information regarding the protein secondary structure. The CD spectrum of HSA displays two negative bands: one at 208 nm and other at 222 nm, which are characteristic of the α-helix structure [52]. Figure 7 shows the CD spectra for HSA in PBS medium, without and in the presence of **5e** at 310K. In this figure, the ellipticity signal was less negative after the addition of the potential anti-*Leishmania amazonensis* agent, suggesting a possible perturbation on the secondary structure of the albumin. Taking into account the quantitative values at 208 and 222 nm for HSA (57.1% and 53.5%, respectively) as well as for HSA:**5e** (55.2% and 52.2%, respectively), a discrete variation in the α-helix percentage for HSA can be observed. These results demonstrated that **5e** is able to induce slight conformational changes on HSA [53], which is consistent with the results described above employing SF spectroscopy.

### 3.5. Molecular Docking Analysis for HSA:***5e***

**Figure 8:**
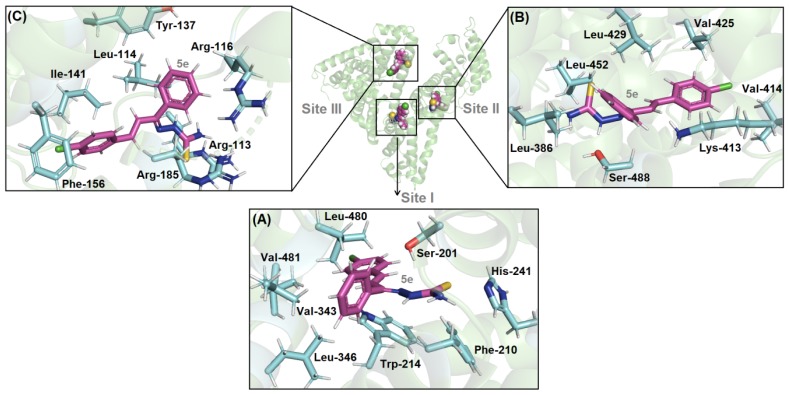
Best docking pose (ChemPLP function) for the interaction HSA:**5e** inside three main binding pockets: (**A**) site I, (**B**) site II and (**C**) site III. Selected amino acid residues are represented as stick in blue, while **5e** structure is represented in pink. Hydrogen, oxygen, nitrogen, sulfur and chloro atoms are represented in white, red, dark blue, yellow and green, respectively.

HSA is a globular protein composed of three homologous α-helical domains (I-III), and each domain contains two subdomains (A and B). There are three main binding pockets in the albumin structure: two located in hydrophobic cavities in subdomains IIA and IIIA (sites I and II, respectively) and one located in an external region in subdomain IB (site III) [35]. In order to suggest the main binding site for the compound **5e** toward serum albumin’s binding pockets, as well as to offer a molecular description (calculate the possible conformation of each ligand) on the binding ability, molecular docking calculations were carried out with Gold 5.6 program. Figure 8 depicts the best docking pose for the ligand inside the three main possible binding sites (I, II and III), and Table 4 shows the main amino acid residues, which can interact with the potential anti-*Leishmania amazonensis* agent.

The docking score of each pose was calculated as the negative of the sum of a series of energy terms involved in the protein-ligand interaction process, so that the more positive the score, is the better the interaction. The highest docking score value for the docking pose of **5e** in the sites I, II and III was 81.5, 59.6 and 65.6, respectively. Since the docking score value was positive for all possible binding sites, there is an indication that the ligands could bind in the three subdomains (not at the same time, according to the experimental number of binding sites: *n* ~ 1.0); however, the highest docking score value was obtained for site I, suggesting subdomain IIA, where Trp-214 residue can be found, as the main binding site. This result is in accordance with the experimental data, which indicated a ground state association between Trp-214 residue and **5e** (see Section 3.3). From the literature, some thiosemicarbazone derivatives were also able to interact inside subdomain IIA (site I) [54,55].

Molecular docking results suggested hydrogen bonding and van der Waals forces as the main intermolecular interaction between **5e** and the amino acid residues presented in the three different binding sites (Table 4). These results are in accordance with the experimental thermodynamic parameters once the cited two main binding forces were detected (see Section 3.3). In addition, it is important to note that according to the experimental Δ*S*° value, the positive parameter could be related to hydrophobic forces and/or desolvation process. According to the docking results, hydrophobic forces were not contributing to the binding ability of compound **5e**, thus, the desolvation process is the phenomenon that could explain the experimental Δ*S*° value. Overall, the theoretical analysis corroborated with the experimental data.

## 4. Conclusions

A novel series of chalcone-thiosemicarbazones were synthesized in reasonable yields. All compounds tested against intracellular amastigotes of *L. amazonensis* were more potent and selective than the reference drug (pentamidine). The most active compound (**5e**) showed an IC_50_ value of 5.22 ± 0.75 µM and the compounds **5a** and **5f** had a selectivity index higher than 10 and an IC_50_ value below of 10 µM, showing that both are promising anti-leishmanial hits. The interaction between HSA and **5e** (the selected compound as a model to study preliminary pharmacokinetic evaluation) indicated a spontaneous, moderate, enthalpically and entropically driven association. The binding occurs via a ground state interaction (static process) and does not perturb significantly both microenvironment and secondary structure of albumin. According to the thermodynamic parameters and molecular docking results, the main intermolecular forces involved in the binding process towards serum albumin are hydrogen bonding and van der Waals interactions. Overall, the results of the study of their antiparasitic effects against *L. amazonensis*, as well as the binding ability toward HSA revealed the chalcone-thiosemicarbazone class as a novel prototype for drug development against leishmaniasis.

## Figures and Tables

**Table 1:** In vitro anti-leishmanial activity of chalcone-thiosemicarbazones against promastigotes, axenic amastigotes, and intracellular amastigotes of *L. amazonensis* after 48 h of culture, and their macrophage cytotoxicity and selectivity index.

Compound	Promastigotes IC_50_^a^ (µM)	Axenic Amastigotes IC_50_ (µM)	Intracellular Amastigotes IC_50_ (µM)	Macrophages LD_50_^b^ (µM)	Selectivity Index (SI)
**5a**	14.68 ± 0.31	6.88 ± 1.09	3.40 ± 0.28	40.98 ± 1.12	15.05
**5b**	14.80 ± 1.92	7.08 ± 1.03	5.95 ± 0.61	56.35 ± 0.78	9.47
**5c**	n.a. ^c^	n.d. ^d^	n.d.	n.d.	−
**5d**	13.09 ± 1.85	5.57 ± 1.15	> 3.13	35.47 ± 1.38	−
**5e**	5.22 ± 0.75	3.19 ± 1.20	4.47 ± 0.42	32.48 ± 1.81	7.27
**5f**	12.52 ± 0.99	4.24 ± 1.07	3.88 ± 0.24	44.24 ± 4.21	11.40
**5g**	n.a.	n.d.	n.d.	n.d.	−
Pentamidine	4.90 ± 0.60	12.29 ± 1.17	11.12 ± 1.98	25.85 ± 4.06	2.32

^a^ IC_50_: half maximal inhibitory concentration; ^b^ LD_50_: median lethal dose; ^c^ n.a.: no active; ^d^ n.d.: not determined.

**Table 2:** Electronic, steric and lipophilic parameters: Hammett constant (σ_p_), Taft constant (Es), molecular refractivity (MR), and Hansh constant (π_m_).

Compound	4-X	σ_p_^a^	Es ^b^	MR ^b^	π ^b^
**5a**	H	0.00	0.00	0.00	0.00
**5b**	CH_3_	−0.14	−0.25	0.57	0.56
**5c**	CN	0.71	−0.51	0.63	−0.57
**5d**	F	0.15	−0.46	0.10	0.14
**5e**	Cl	0.34	−0.97	0.60	0.71
**5f**	Br	0.26	−1.16	0.89	0.86
**5g**	NO_2_	0.81	−1.01	0.74	−0.28

^a^ Isaacs, N. [45]; ^b^ Hoekman, D. [46].

**Table 3:** Binding constant values for the interaction between HSA and **5e** at four temperatures.

T (K)	*K_SV_* (×10^4^) (M^−1^) ^a^	*k_q_* (×10^12^) (M^−1^s^−1^) ^a^	*K_a_* (×10^4^) (M^−1^) ^b^	*n* ^b^	Δ*H*° (kJmol^−1^) ^c^	Δ*S*° (×10^−2^) (kJmol^−1^K^−1^) ^c^	Δ*G*° (kJmol^−1^) ^c^
289	2.48 ± 0.52	4.15	5.16 ± 0.04	1.05 ± 0.01	−5.79 ± 0.27	7.02 ± 0.09	−26.1
296	2.49 ± 0.50	4.16	4.85 ± 0.06	1.07 ± 0.01			−26.6
303	2.59 ± 0.51	4.34	4.57 ± 0.06	1.06 ± 0.01			−27.1
310	2.75 ± 0.33	4.60	4.39 ± 0.04	1.05 ± 0.01			−27.6

^a^*r*^2^: 0.9996–0.9987; ^b^*r*^2^: 0.9998–0.9996; ^c^*r*^2^: 0.9978.

**Table 4:** Molecular docking results for the interaction HSA:**5e** in the sites I, II and III.

Sites	Amino Acid Residues	Interaction	Distance (Å)
	Phe-210	Van der Waals	2.60
	Trp-214	Van der Waals	3.00
	Ser-201	Hydrogen bonding	2.80
I	His-241	Van der Waals	3.60
	Val-343	Van der Waals	3.50
	Leu-346	Van der Waals	2.40
	Leu-480	Van der Waals	2.70
	Val-481	Van der Waals	2.70
	Leu-386	Van der Waals	3.20
	Lys-413	Van der Waals	3.10
	Val-414	Van der Waals	2.90
II	Val-425	Van der Waals	2.20
	Leu-429	Van der Waals	2.90
	Leu-452	Van der Waals	3.60
	Ser-488	Hydrogen bonding	1.80
	Arg-113	Hydrogen bonding	3.30
	Leu-114	Van der Waals	2.40
	Arg-116	Van der Waals	2.40
III	Tyr-137	Van der Waals	2.40
	Ile-141	Van der Waals	2.70
	Phe-156	Van der Waals	3.70
	Arg-185	Hydrogen bonding	3.80

## Supplementary Materials

The following are available online at https://www.mdpi.com/2218-273X/9/11/643/s1, Figures S1–S19: all spectra for the characterization of the synthetic compounds **5a**–**5g**. Figure S20: Double logarithmic plots for the interaction between HSA:**5e** at 289 K, 296 K, 303 K, and 310 K. Figure S21: Van’t Hoff plot for the interaction between HSA:**5e** at four different temperatures.
