# Supplementary material for: Evaluation of Novel Chalcone-Thiosemicarbazones Derivatives as Potential Anti-Leishmania amazonensis Agents and Its HSA Binding Studies
Source: Biomolecules. 2019 Oct 23;9(11):643. doi: 10.3390/biom9110643 (PMC6920794; doi:10.3390/biom9110643)

Supplementary Material

Evaluation of Novel Chalcone-Thiosemicarbazones Derivatives as Potential Anti-*Leishmania amazonensis* Agents and its HSA binding studies

Edinéia Pastro Mendes [1,2], Carla Marins Goulart [1], Otávio Augusto Chaves [1,3], Viviane dos S. Faiões [4], Marilene M. Canto-Carvalho [4], Gerzia C. Machado [4], Eduardo Caio Torres-Santos [4,*], and Aurea Echevarria [1,*]

[1]Instituto de Química, Departamento de Química Orgânica, Universidade Federal Rural do Rio de Janeiro, 23.890-000 Seropédica-RJ, Brazil; edinea@bio.fiocruz.br (E.P.M.); cmarinsgoulart@gmail.com (C.M.G.)

[2]Programa de Pós-graduação em Ciência, Tecnologia e Inovação em Agropecuária, Universidade Federal Rural do Rio de Janeiro, Seropédica,23890-000 Seropédica-RJ, Brazil;

[3]Instituto SENAI de Inovação em Química Verde, Rua Morais e Silva N°53, Bloco 09, 20271-030 Maracanã-RJ, Brazil; oachaves@firjan.com.br (O.A.C.)

[4]Laboratório de Bioquímica de Tripanosomatídeos, Instituto Oswaldo Cruz, FIOCRUZ, 21040-900 Rio de Janeiro-RJ, Brazil; faioes@gmail.com (V.S.F.); mcantoca@ioc.fiocruz.br (M.M.C.-C.); machado@ioc.fiocruz.br (G.C.M.); ects@ioc.fiocruz.br (E.C.T.-S.)

*Corresponding authors:

E-mail: echevarr@ufrrj.br Tel/fax: (55) 21 2682-1172 (A. Echevarria)

E-mail: ects@ioc.fiocruz.br Tel/fax: (55) 21 3865-8247 (E.C. Torres-Santos)

Some Representative Spectra

Figure S1. IR spectrum of 1,3-diphenylprop-2-en-1-one thiosemicarbazone (**5a**)

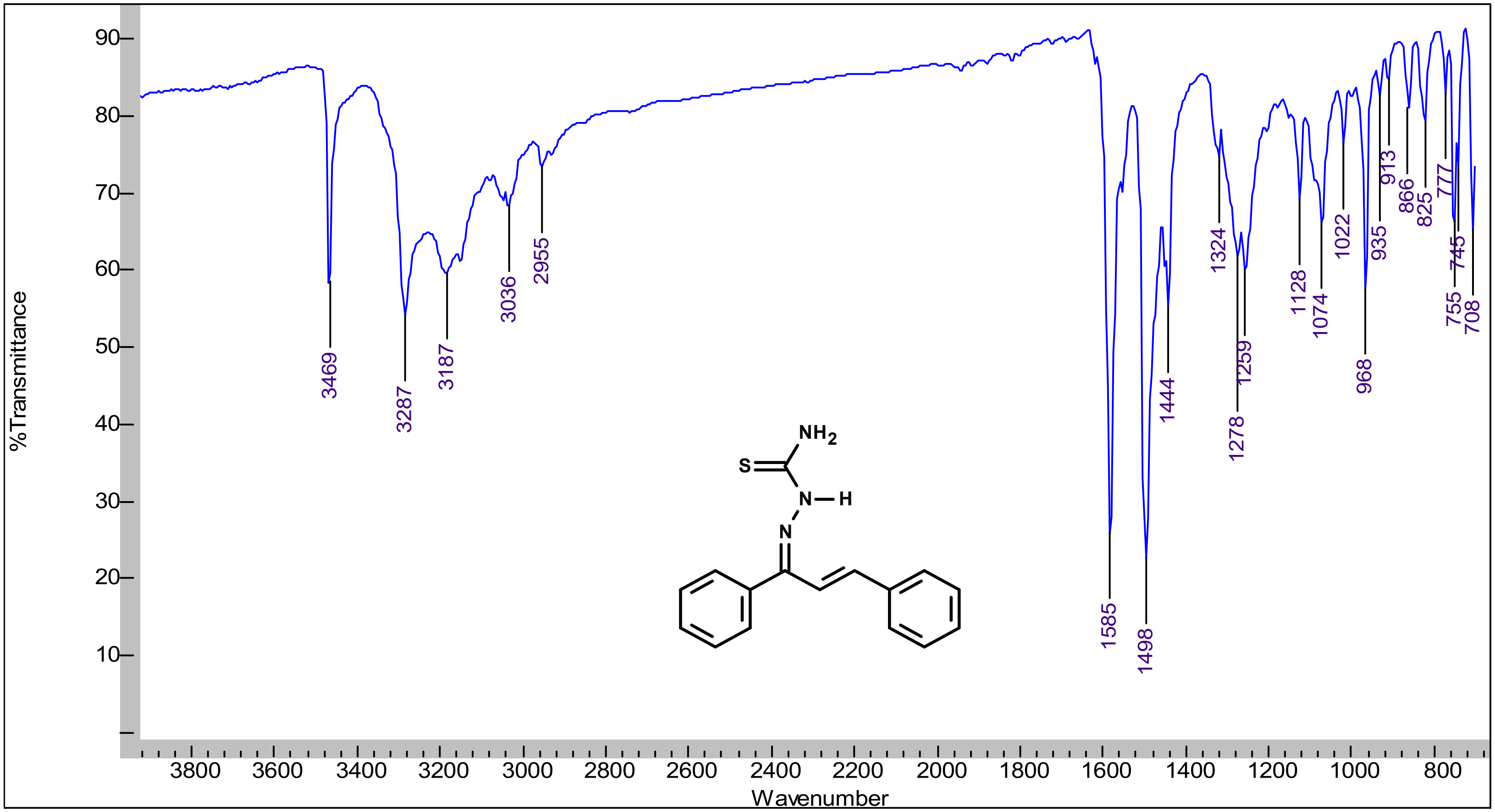

Figure 2. ^{1}H NMR spectrum of 1,3-diphenylprop-2-en-1-ona thiosemicarbazone (**5a**).

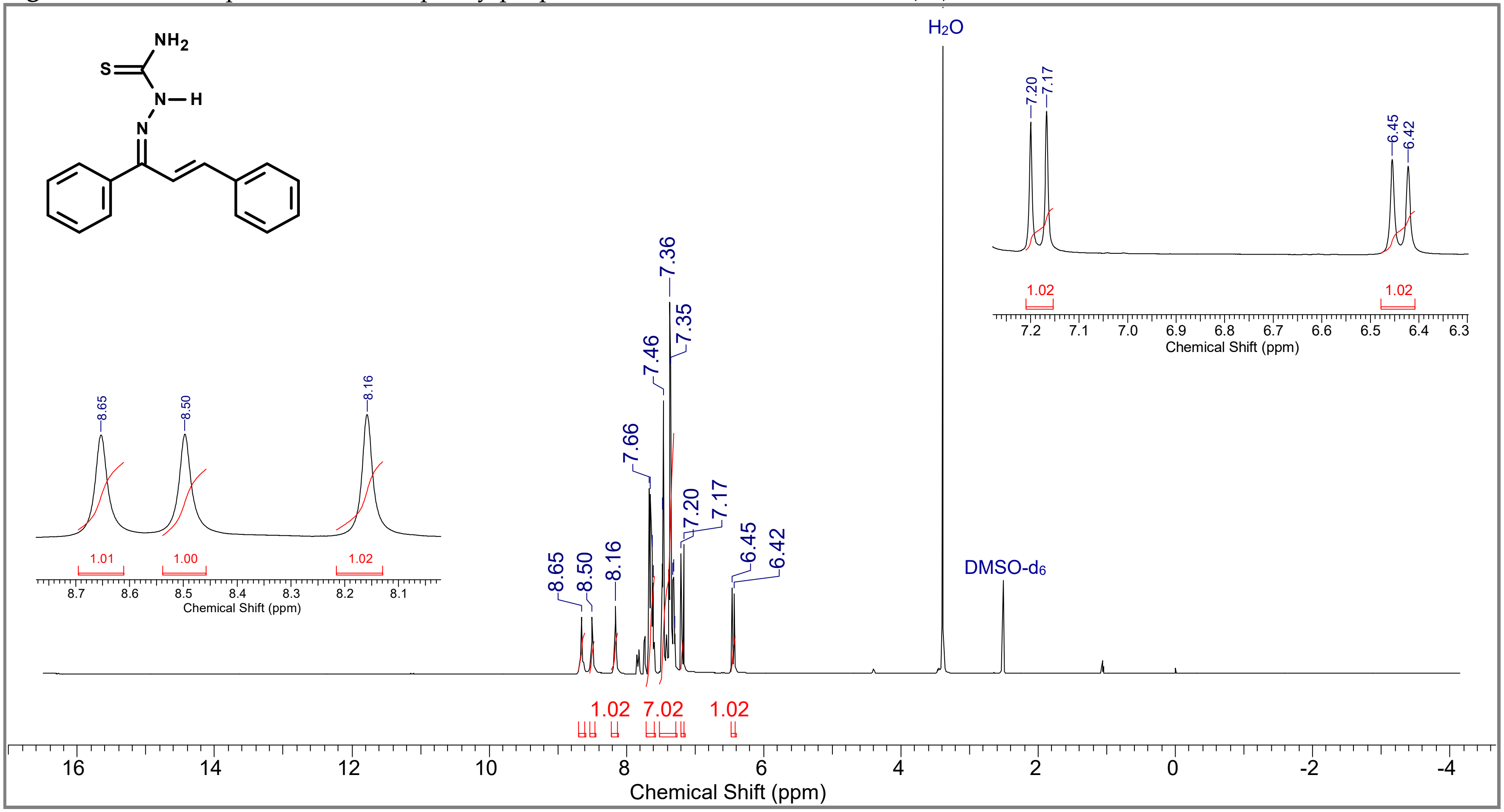

Figure S3. ^{13}C NMR spectrum of 1,3-diphenylprop-2-en-1-one thiosemicarbazone (**5a**)

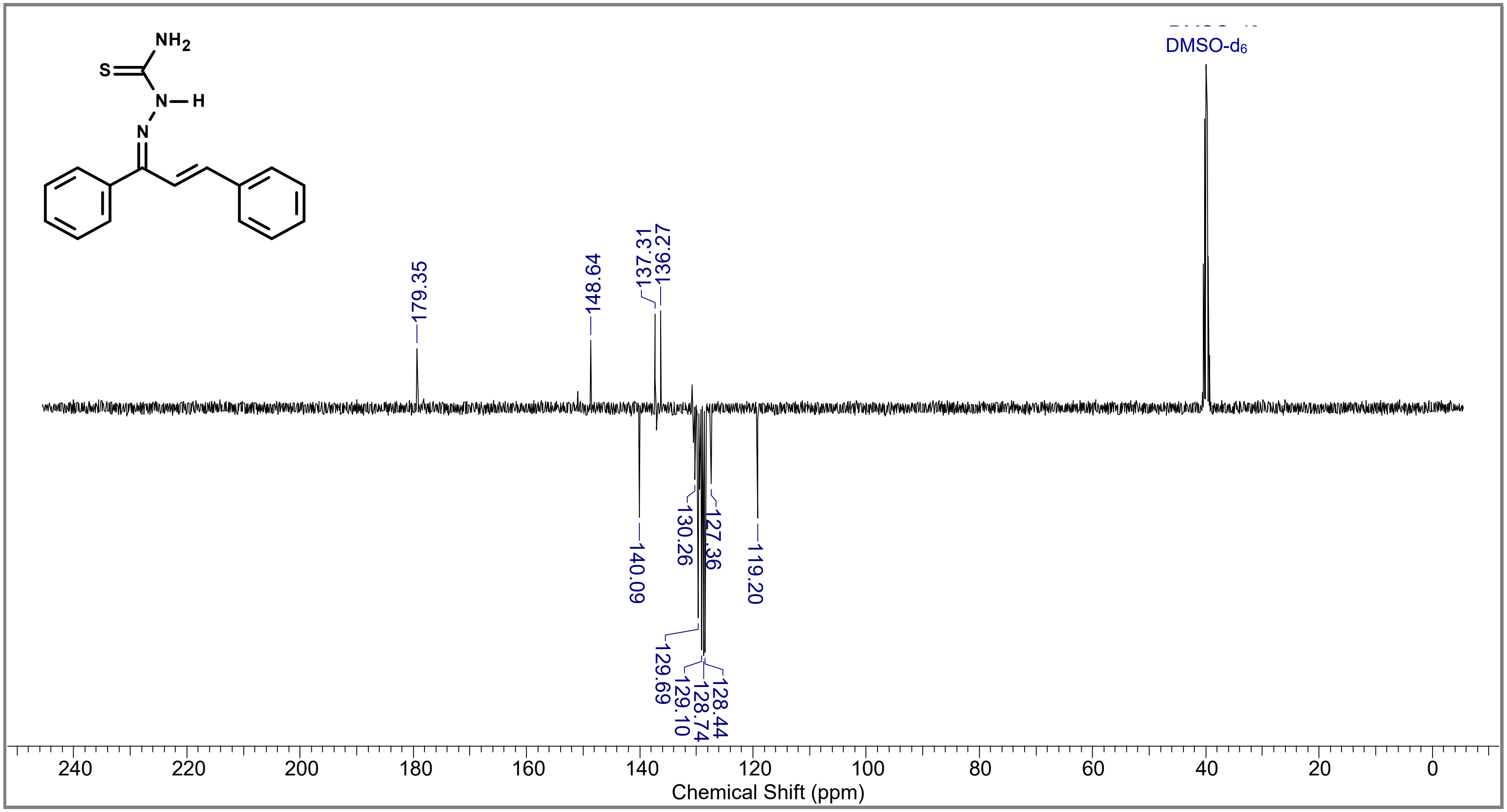

Figure S4. IR spectrum of 3-(4′-methylphenyl)-1-phenylprop-2-en-1-one thiosemicarbazone (**5b**)

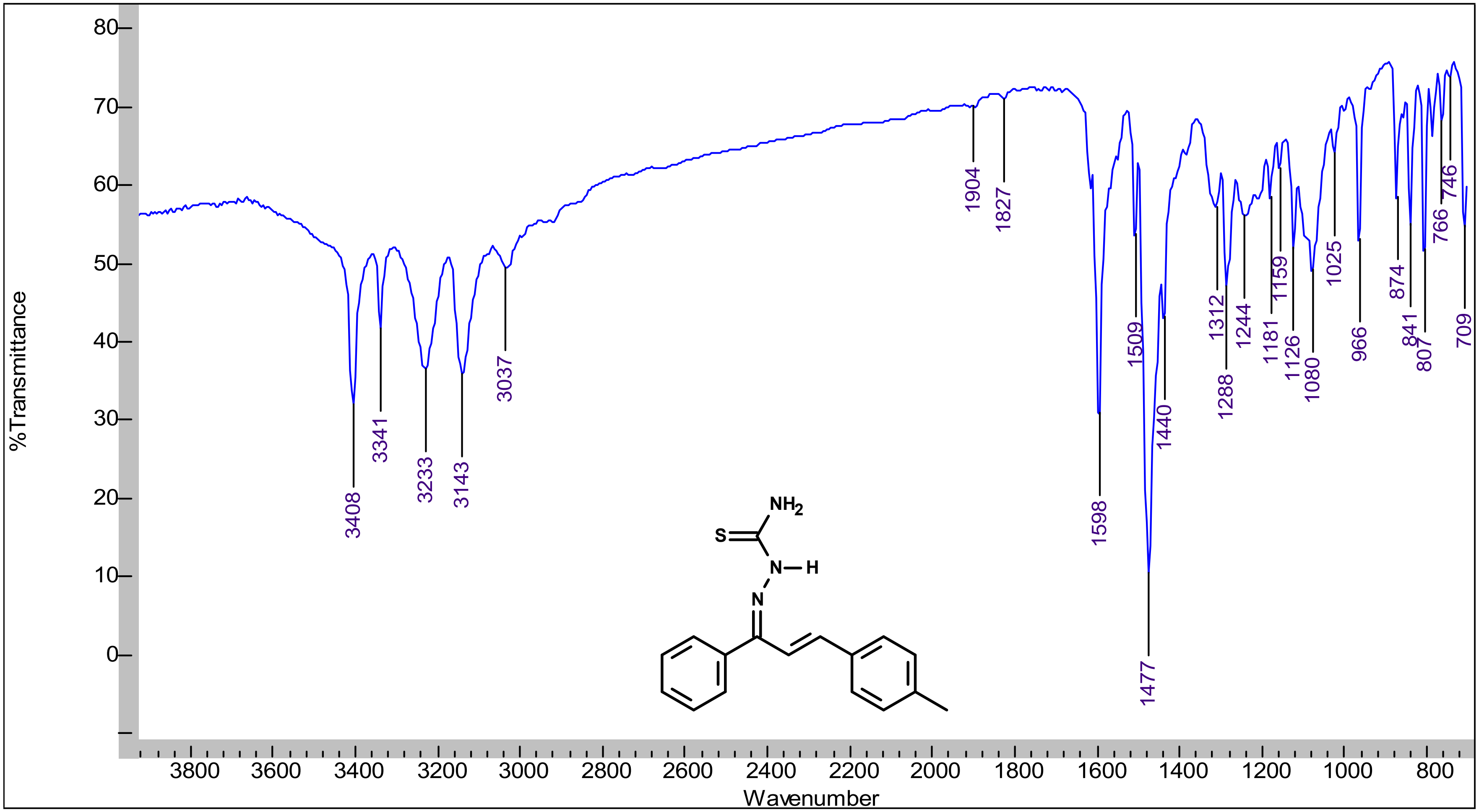

Figure S5. ^{13}C NMR-DEPTQ spectrum of 3-(4′-methylphenyl)-1-phenylprop-2-en-1-one thiosemicarbazone (**5b**)

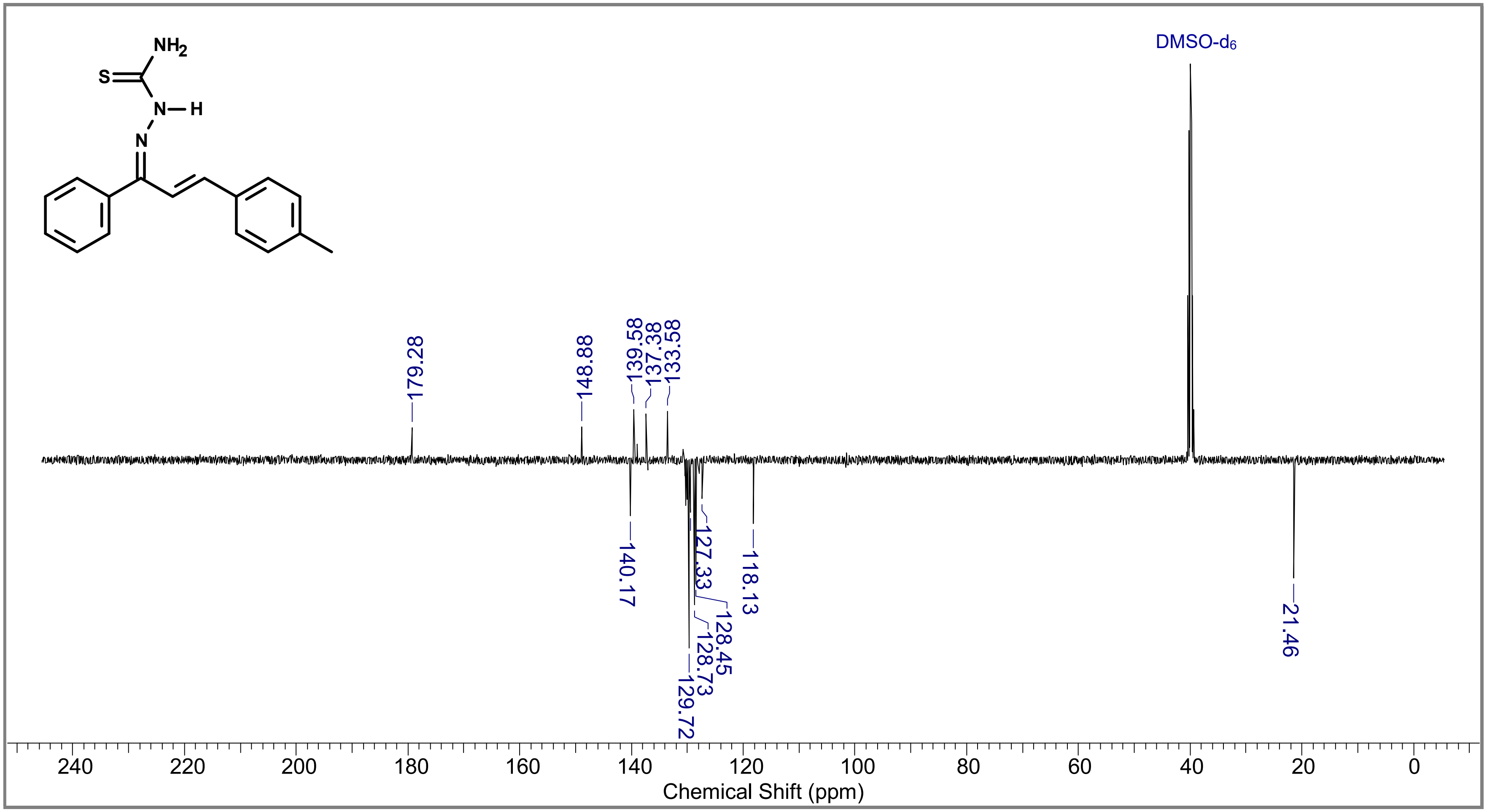

Figure S6. IR spectrum of 3-(4′-cianophenyl)-1-phenylprop-2-en-1-one thiosemicarbazone (**5c**)

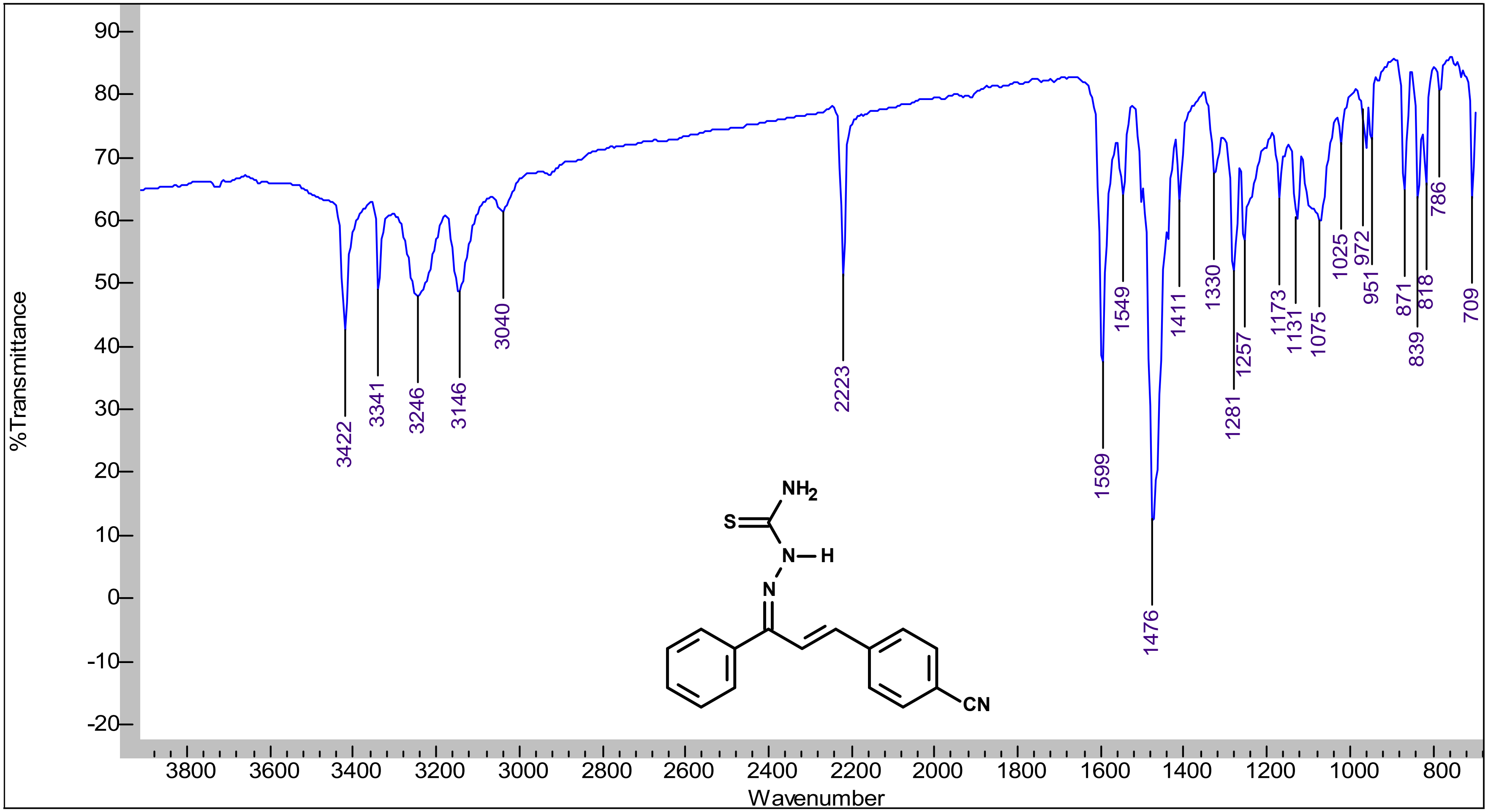

Figure S7. ^{1}H NMR spectrum of 3-(4′-cianophenyl)-1-phenylprop-2-en-1-one thiosemicarbazone (**5c**)

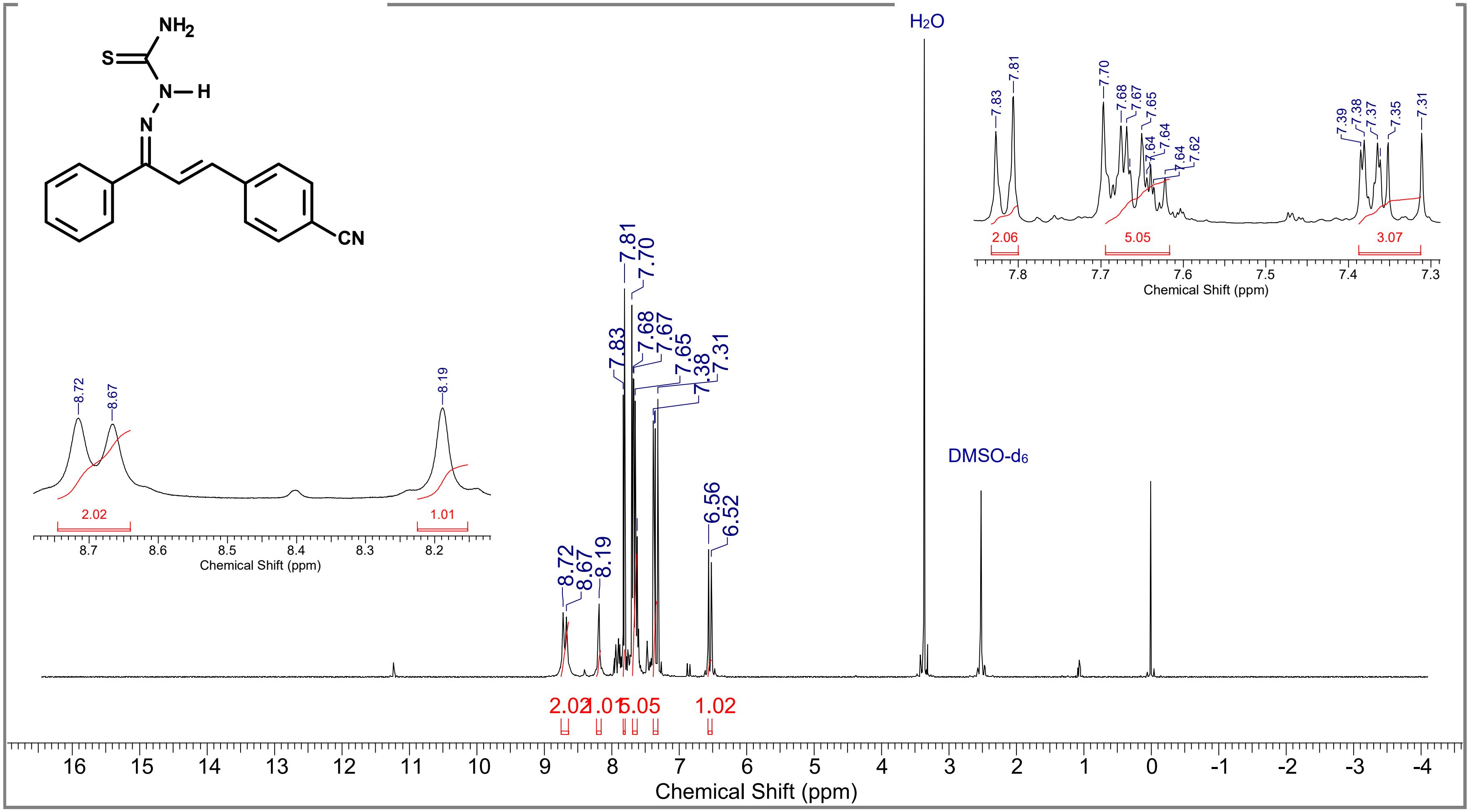

Figure S8. ^{13}C NMR-DEPTQ spectrum of 3-(4′-cianophenyl)-1-phenylprop-2-en-1-one thiosemicarbazone (**5c**)

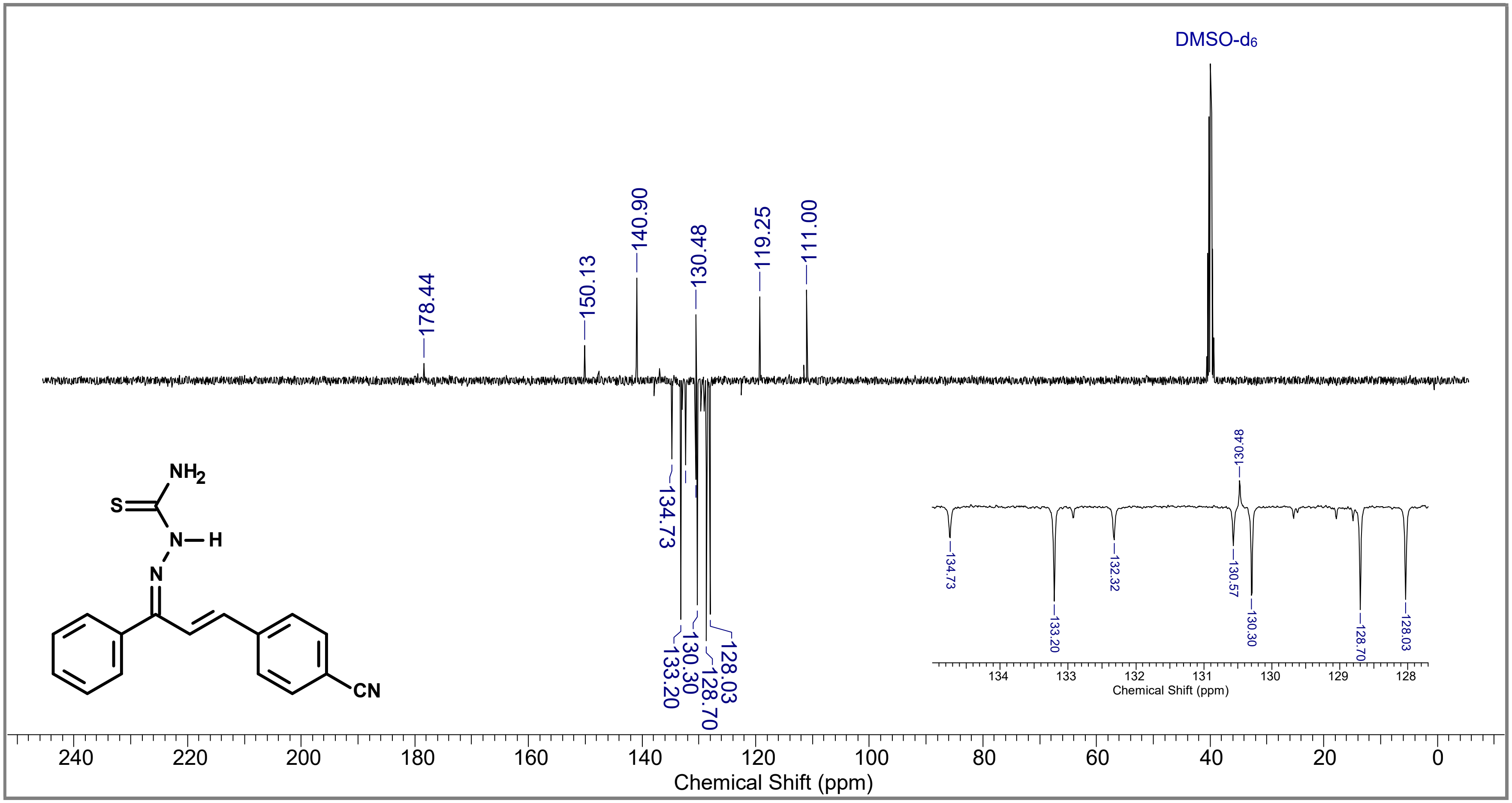

Figure S9. IR spectrum of 3-(4′-fluorphenyl)-1-phenylprop-2-en-1-one thiosemicarbazone (**5d**)

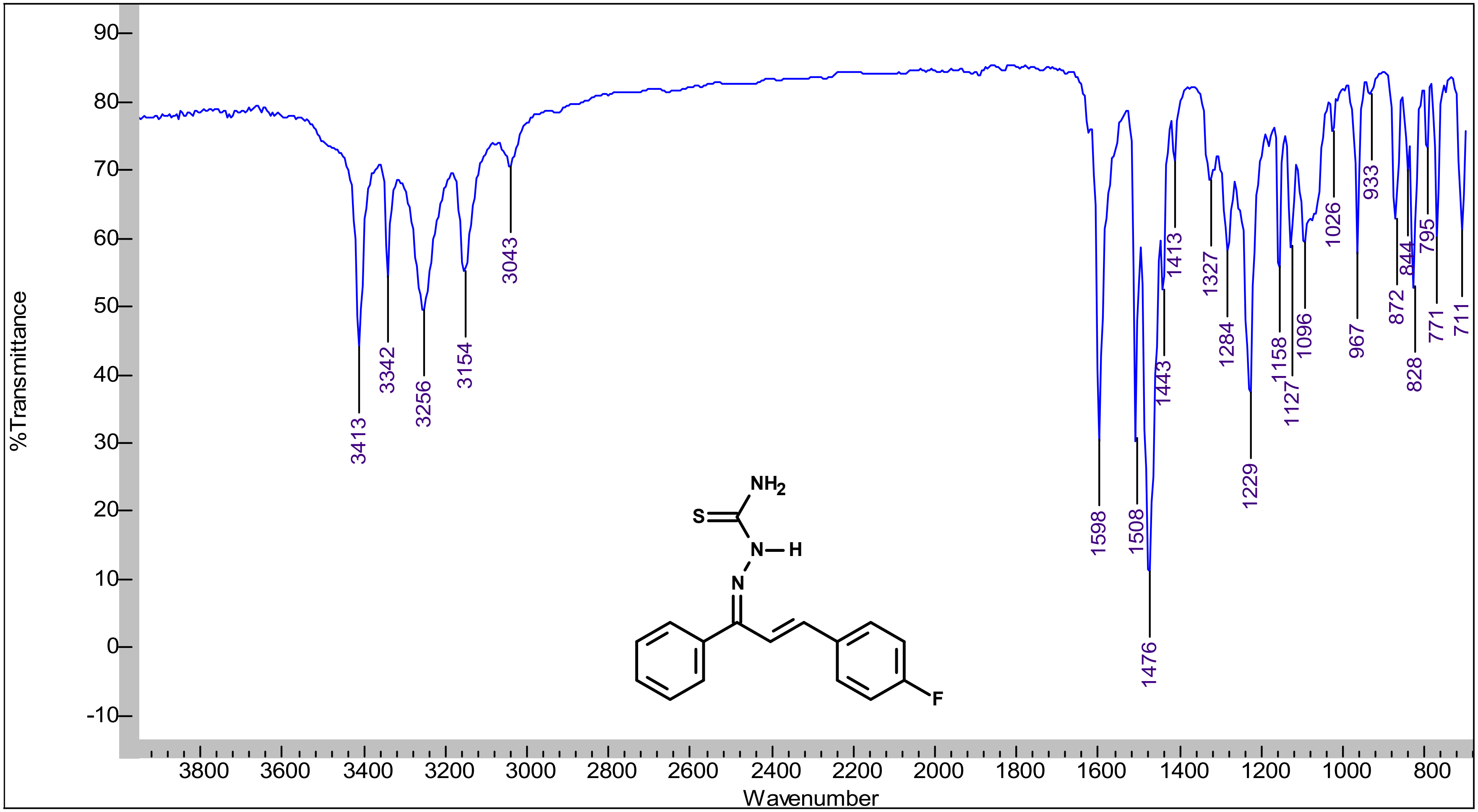

Figure S10. ^{1}H NMR spectrum of 3-(4′-fluorphenyl)-1-phenylprop-2-en-1-one thiosemicarbazone (**5d**)

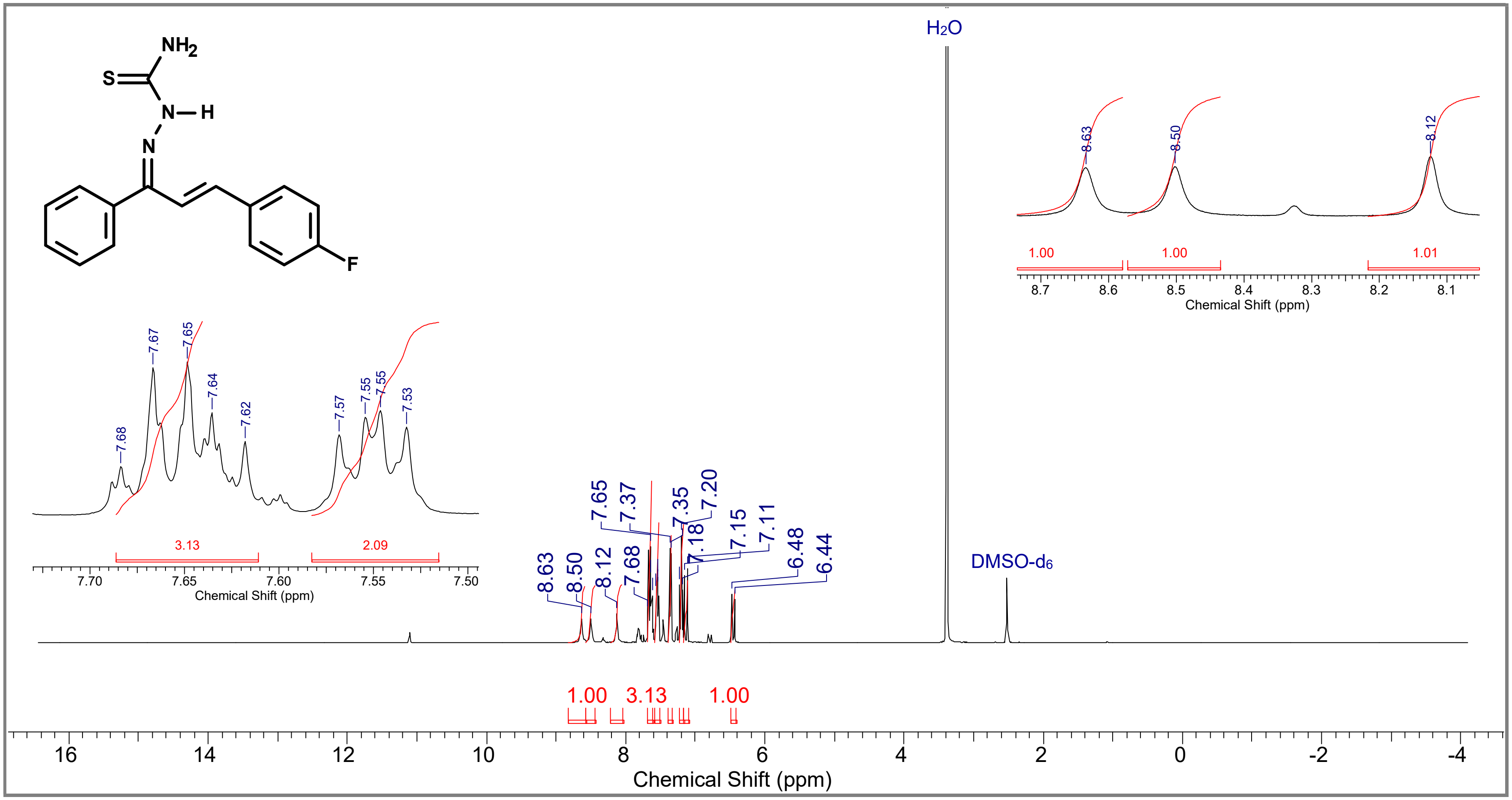

Figure S11. ^{13}C NMR-DEPTQ spectrum of 3-(4′-fluorphenyl)-1-phenylprop-2-en-1-one thiosemicarbazone (**5d**)

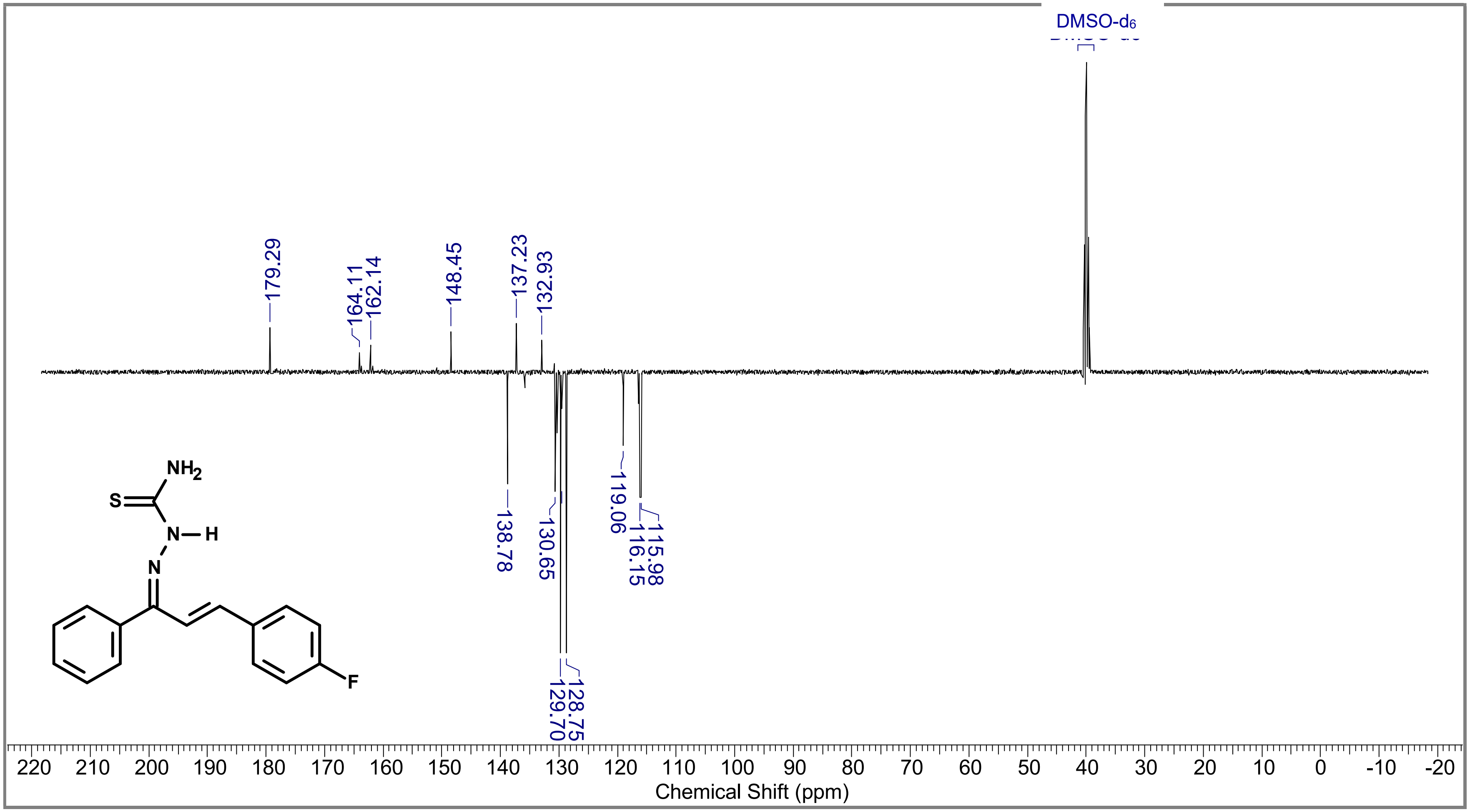

Figure S12. IR spectrum of 3-(4′-clorophenyl)-1-phenylprop-2-en-1-one thiosemicarbazone (**5e**)

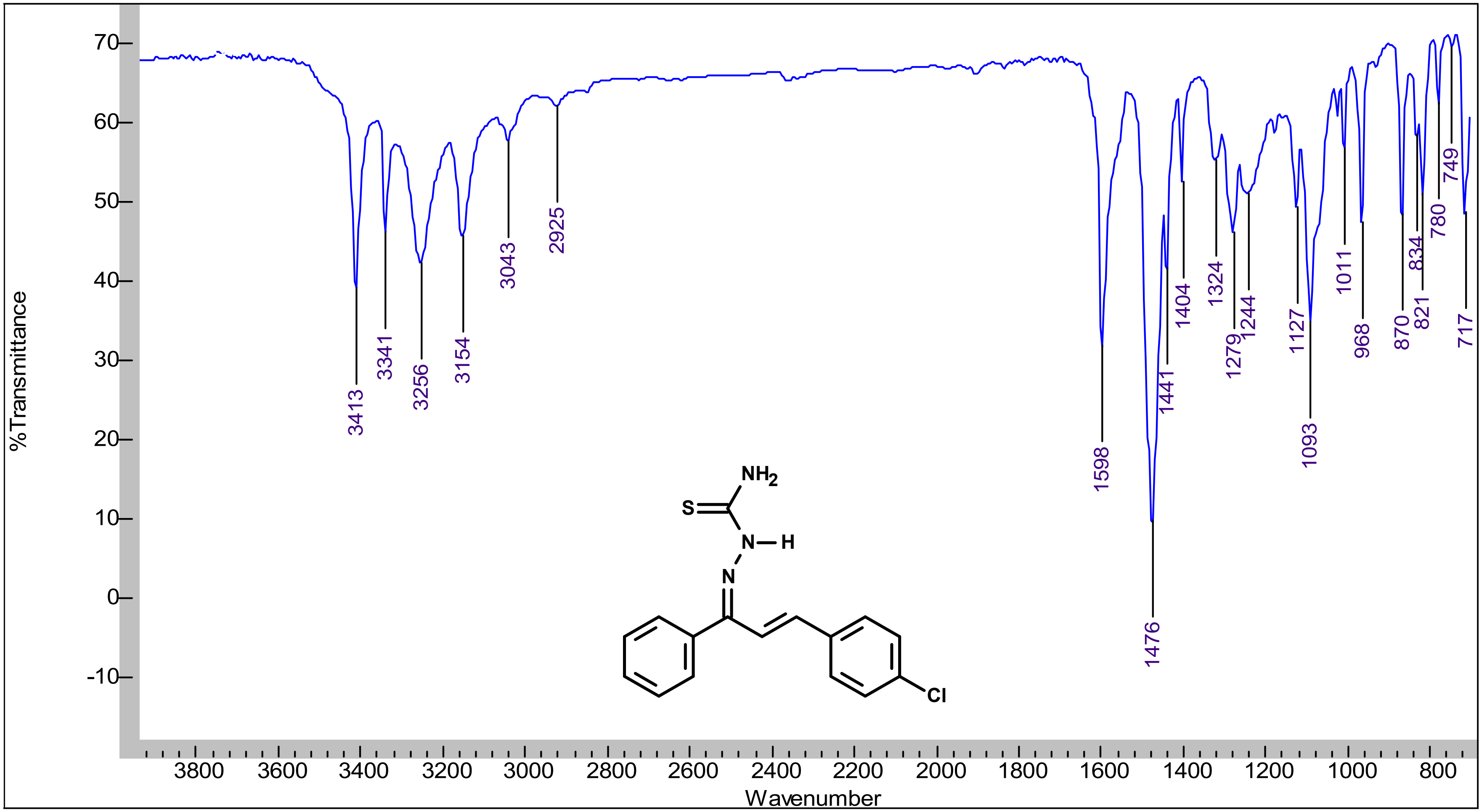

Figure S13. ^{1}H NMR spectrum of 3-(4′-clorophenyl)-1-phenylprop-2-en-1-one thiosemicarbazone (**5e**)

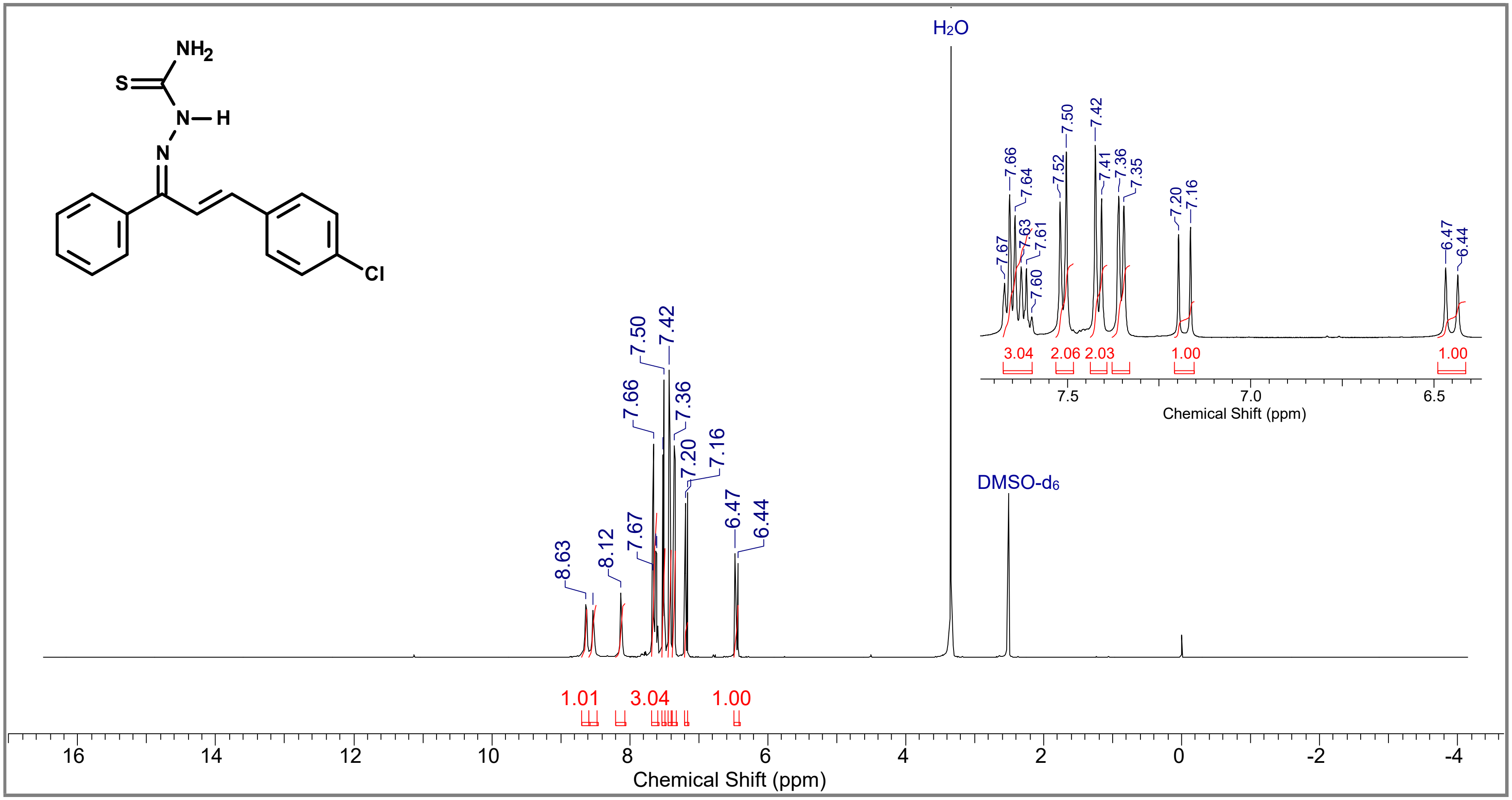

Figure S14. ^{13}C NMR-DEPTQ spectrum of 3-(4′-clorophenyl)-1-phenylprop-2-en-1-one thiosemicarbazone (**5e**)

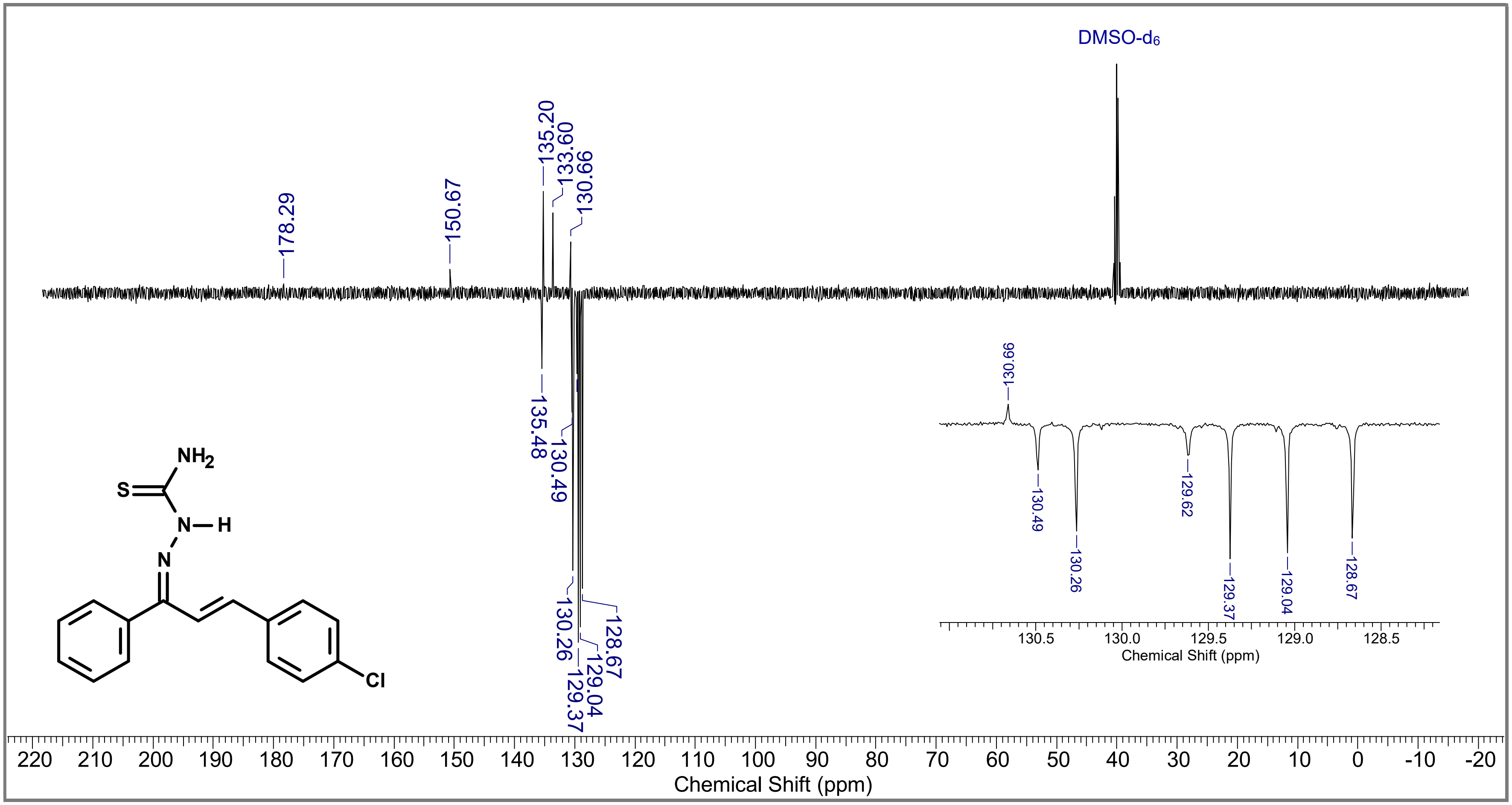

Figure S15. IR spectrum of 3-(4′-bromophenyl)-1-phenylprop-2-en-1-one thiosemicarbazone (**5f**)

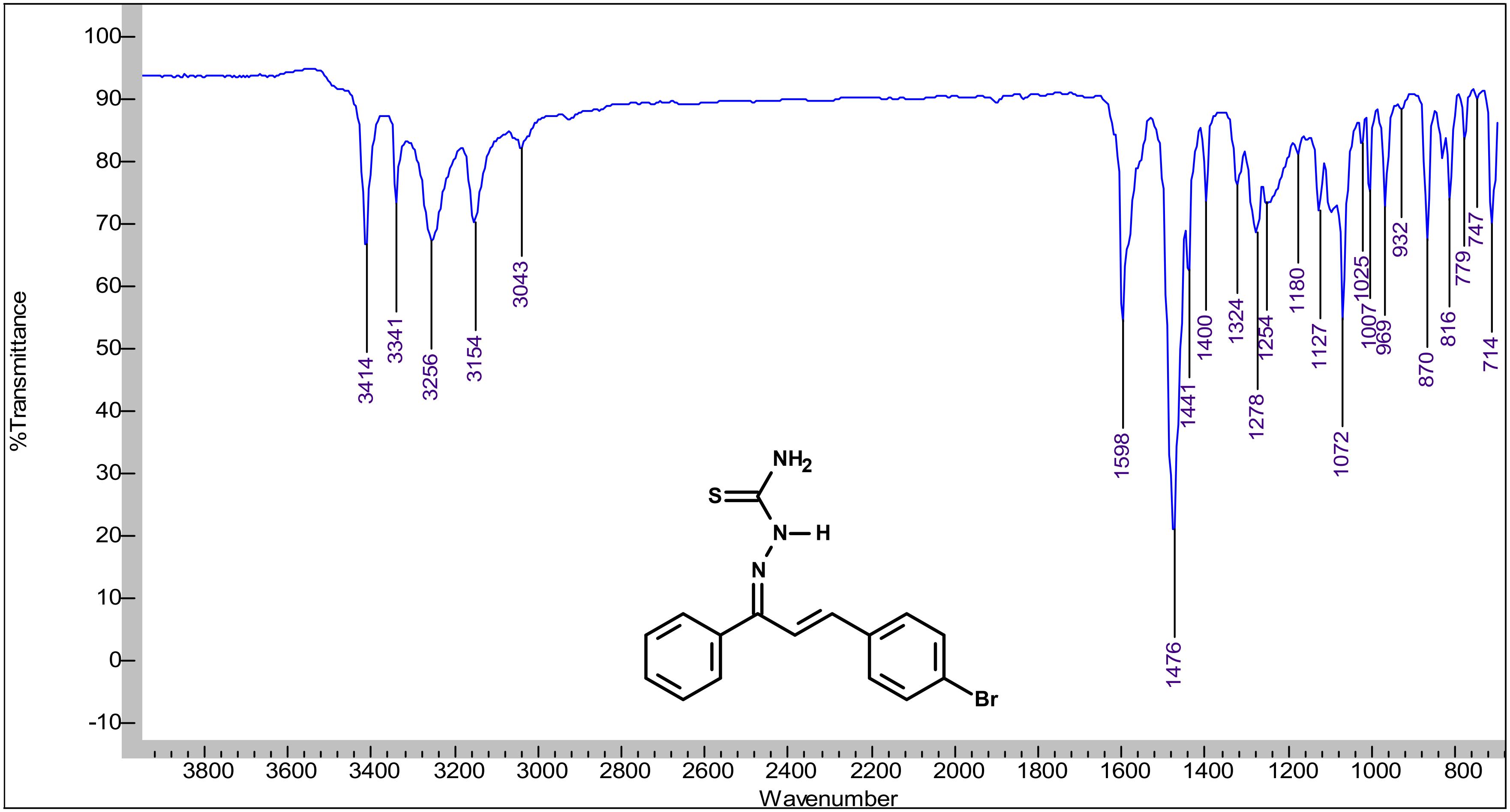

Figure S16. ^{1}H NMR spectrum of 3-(4′-bromophenyl)-1-phenylprop-2-en-1-one thiosemicarbazone (**5f**)

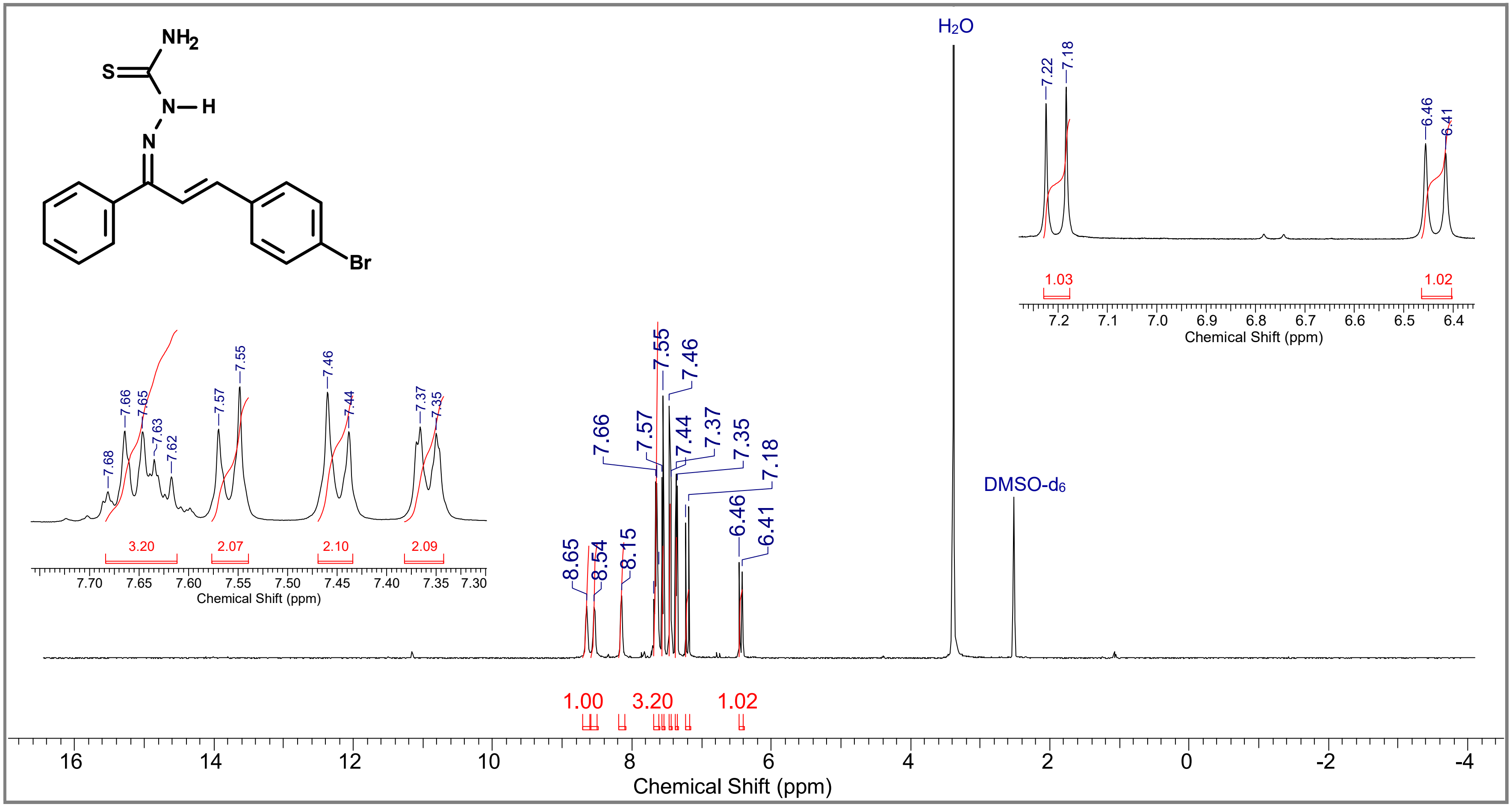

Figure S17. ^{13}C NMR-DEPTQ spectrum of 3-(4′-bromophenyl)-1-phenylprop-2-en-1-one thiosemicarbazone (**5f**)

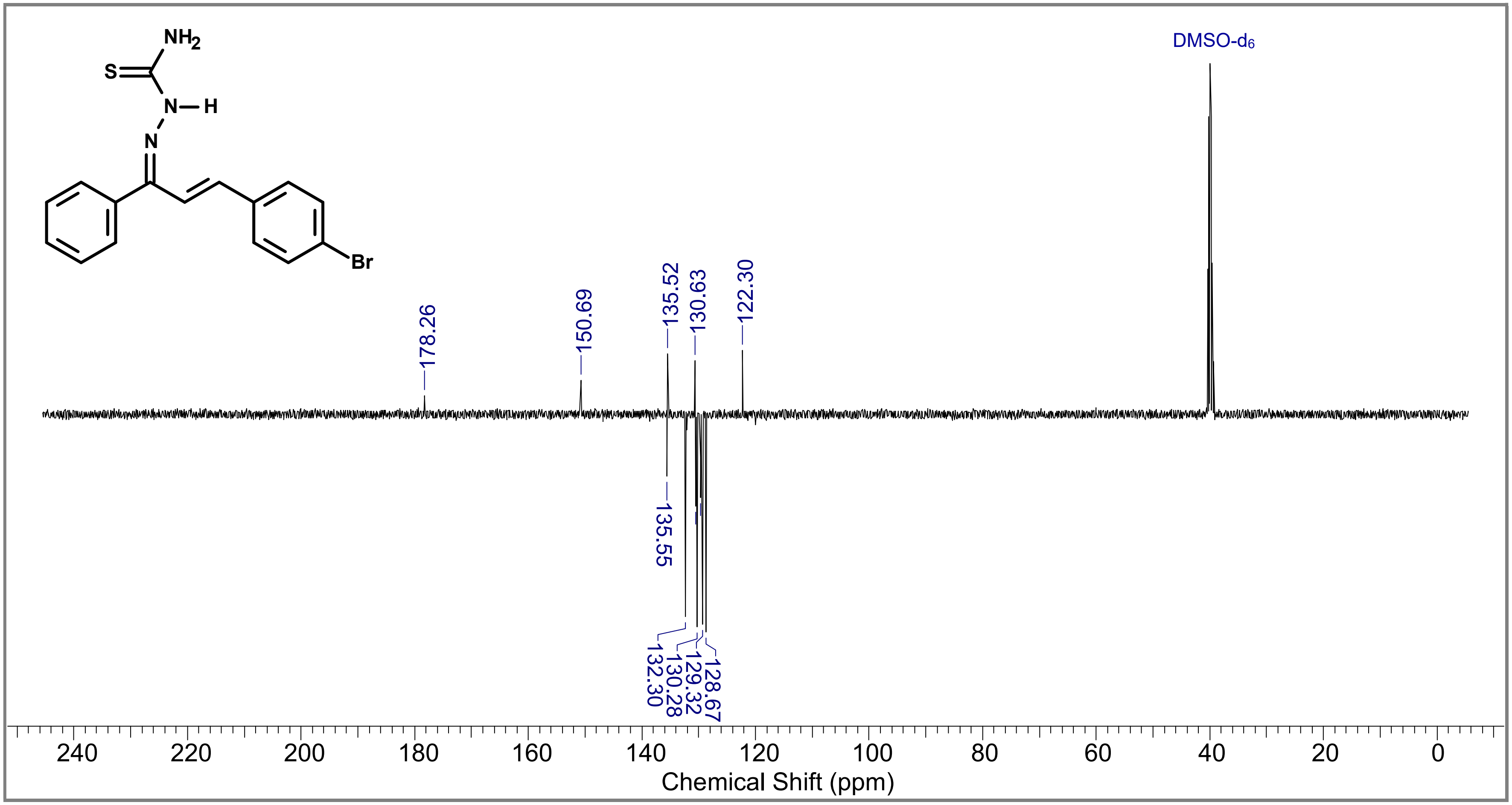

Figure S18. IR spectrum of 3-(4′-nitrophenyl)-1-phenylprop-2-en-1-one thiosemicarbazone (**5g**)

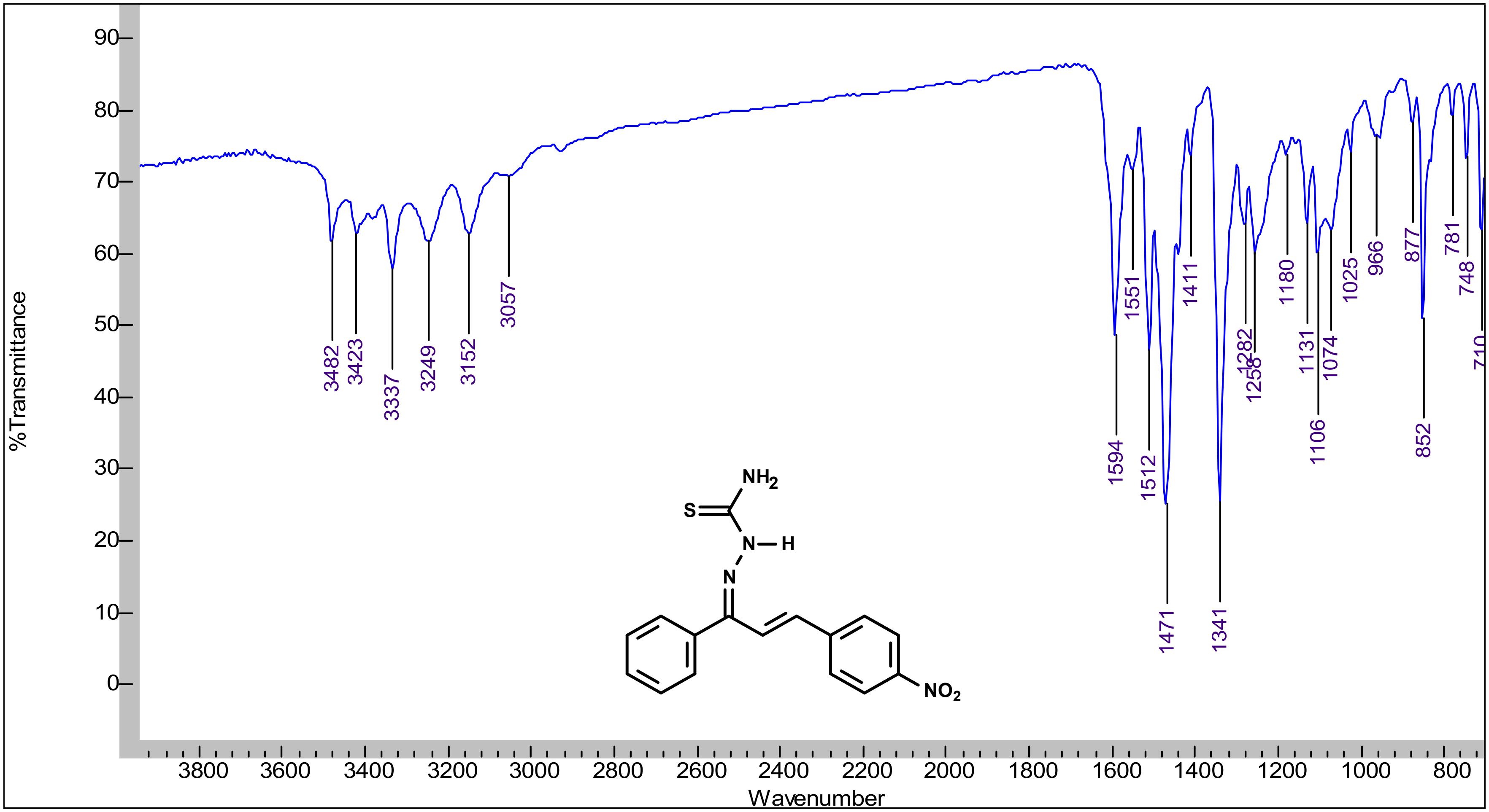

Figure S19. ^{13}C NMR-DEPTQ spectrum of 3-(4′-nitrophenyl)-1-phenylprop-2-en-1-one thiosemicarbazone (**5g**)

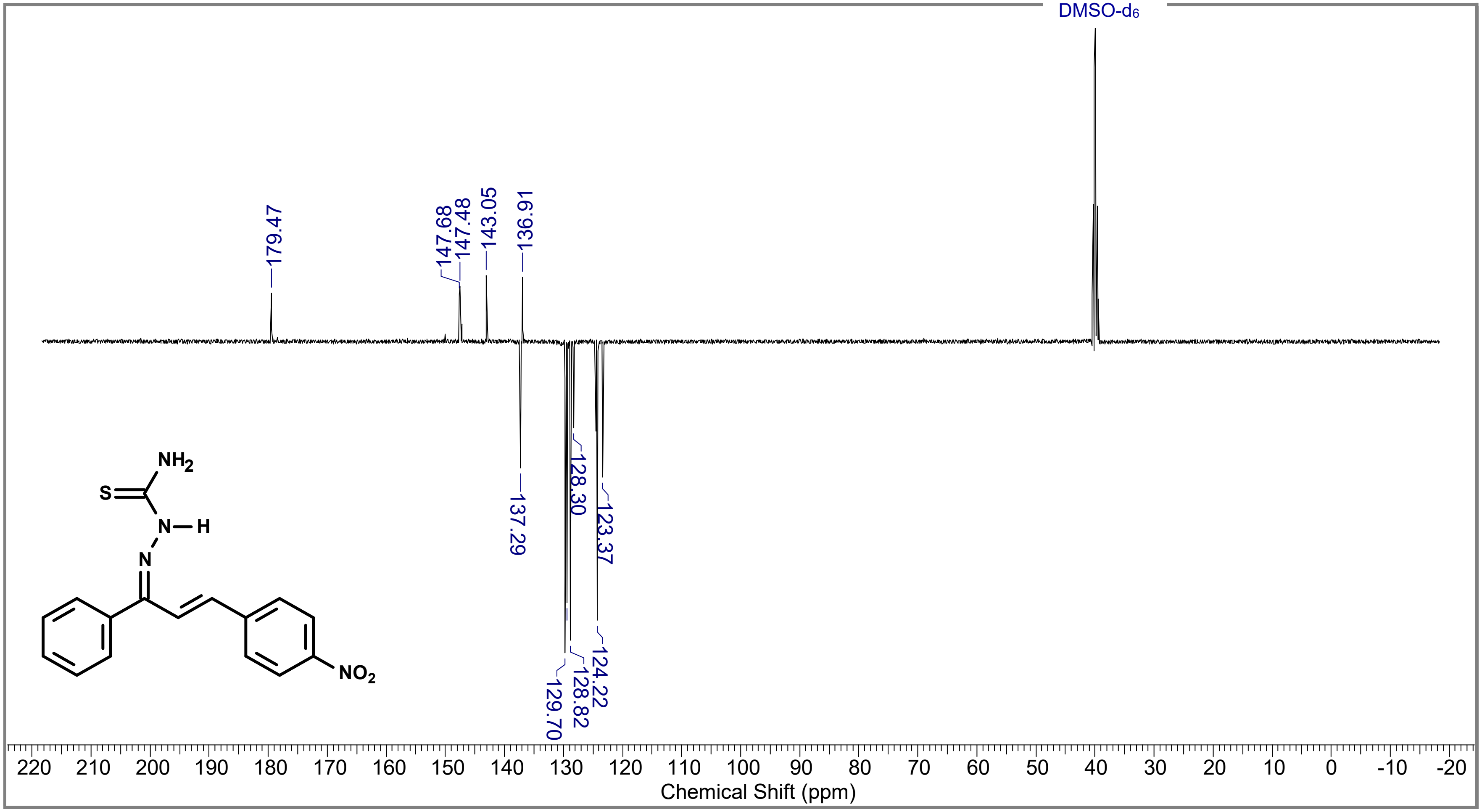

Figure S20. Double logarithmic plots for the interaction between HSA:**5e** at 289K, 296K, 303K, and 310K.

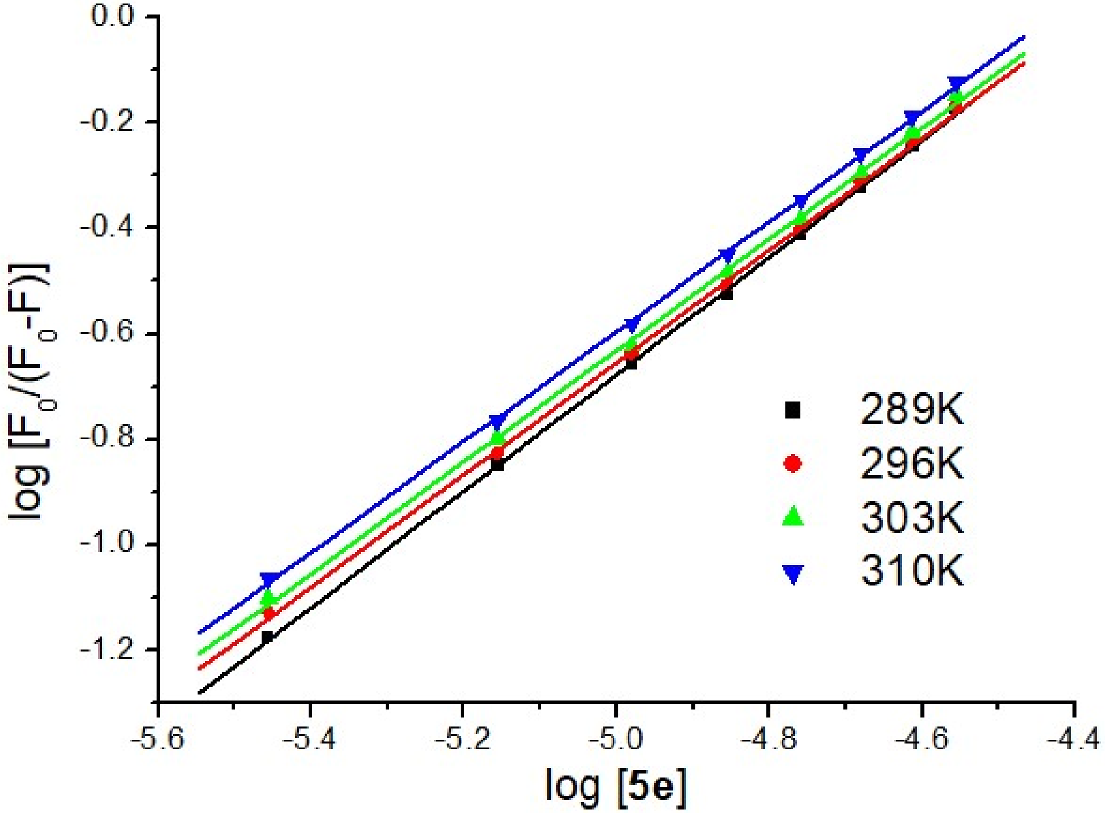

Figure S21. Van't Hoff plot for the interaction between HSA:**5e** at four different temperatures.

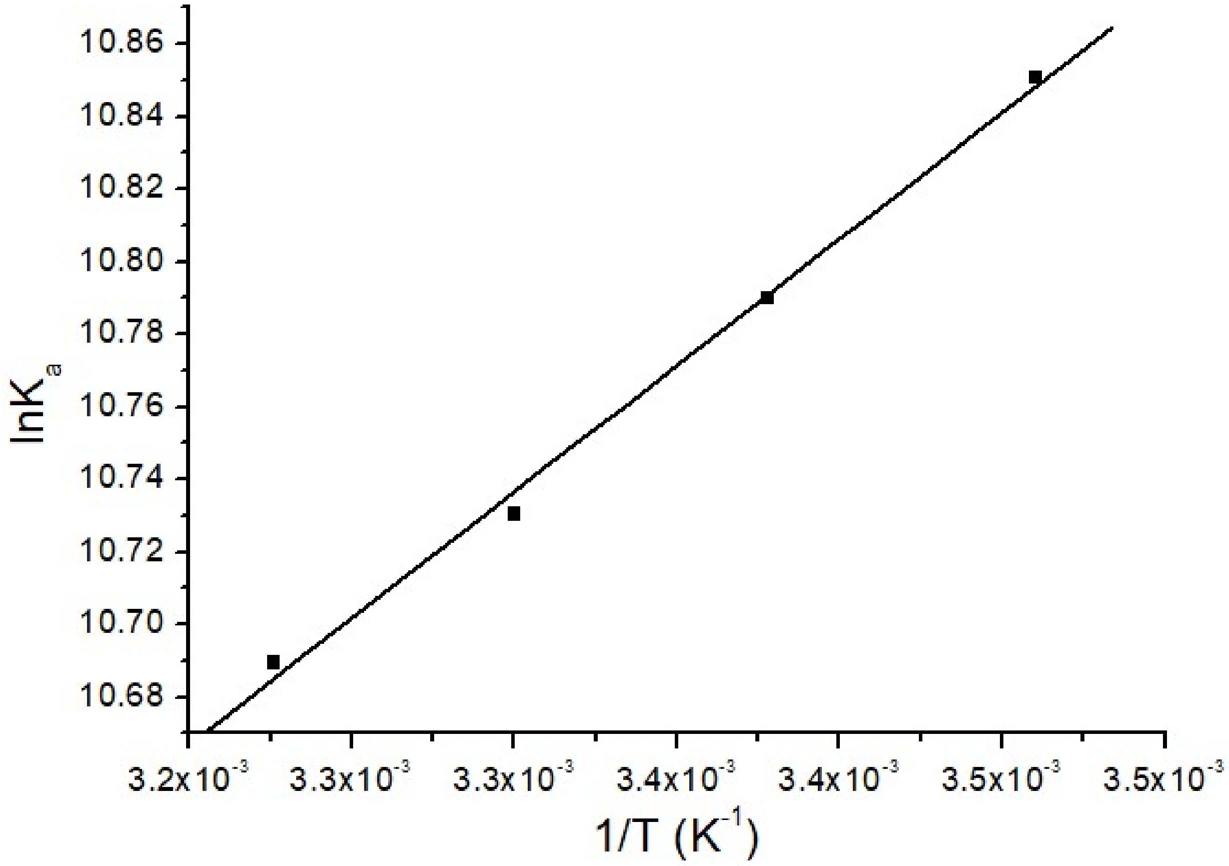

Supplement: Supplementary file 1 [file biomolecules-09-00643-s001.pdf]
